# Probing the binding dynamics of a sunitinib analog with human serum albumin: a combined spectroscopic and molecular docking approach

**DOI:** 10.1039/d6ra07888e

**Published:** 2026-11-02

**Authors:** Sohaila A. Abbas, Eman A. Mostafa, Alshaimaa M. Alian, Ehab F. Elkady, Marwa A. Fouad

**Affiliations:** a Analytical Chemistry Department, Egyptian Drug Authority (EDA) Giza Egypt; b Pharmaceutical Chemistry Department, Faculty of Pharmacy, Cairo University Kasr El-Aini St. Cairo 11562 Egypt marwa.fouad@pharma.cu.edu.eg; c Pharmaceutical Chemistry Department, Faculty of Pharmacy, Al-Baha University Al-Baha 1988 Saudi Arabia; d Pharmaceutical Chemistry Department, School of Pharmacy, New Giza University Giza Egypt

## Abstract

The study of protein binding, particularly interactions involving human serum albumin (HSA), is critical for elucidating drug performance and optimizing therapeutic efficacy. Given the profound impact of drug interactions with HSA on pharmacokinetic profiles, this study aims to characterize the binding interaction between a sunitinib analog (SA), as a promising chemotherapeutic drug candidate and HSA and to gain deeper understanding about the nature of binding interaction, binding constant, number of binding sites, binding location, and the conformational changes resulting from this interaction. The present study employs a comprehensive, multi-model approach. This methodology integrates fluorescence spectroscopy, UV absorption spectroscopy, Fourier-transform, and molecular docking, thereby providing a robust framework for detailed analysis. Fluorescence spectroscopy was implemented at three distinct temperatures to analyze the quenching mechanism and determine the binding constant (*K*_b_). SA can quench the native fluorescence of HSA through a static quenching mechanism. The *K*_b_ value due to SA–HSA interaction was found to be of the order of 10^4^ M^−1^ at 298 K, indicating the moderate affinity of SA to HSA. The negative values of Δ*G*° suggested that the interaction between SA and HSA was spontaneous. According to the thermodynamic analysis, SA binds HSA *via* van der Waals forces and H-bonding interactions. The findings of the ligand-competitive displacement experiment indicate that SA exhibits an affinity for HSA within the subdomain IIIA binding pocket. Analysis of data collected by multiple spectroscopic techniques revealed that SA induces perturbation in the secondary structure of HSA and modifies the polarity of the microenvironment surrounding tryptophan residues.

## Abstract 2

The study of protein binding, particularly interactions involving human serum albumin (HSA), is critical for elucidating drug performance and optimizing therapeutic efficacy.

## Graphical abstract

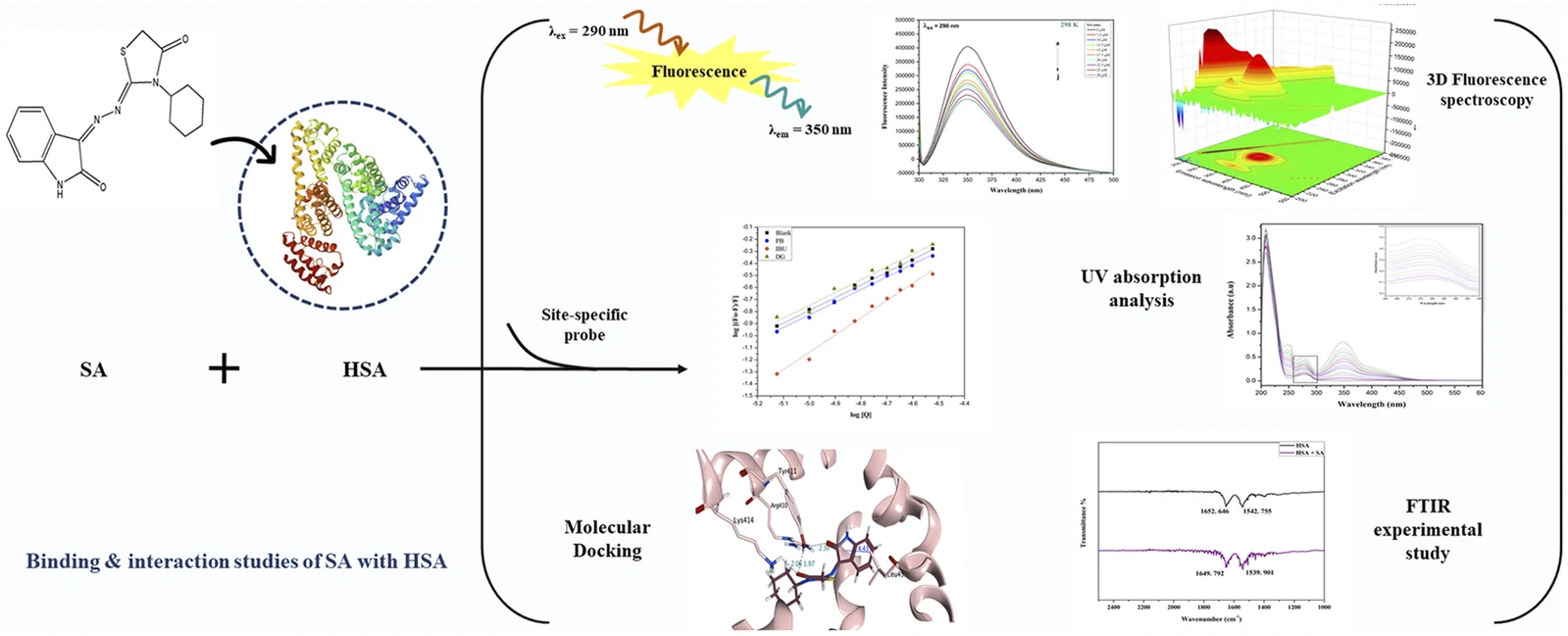

## 1. Introduction

The therapeutic efficacy of a pharmaceutical compound is primarily determined by its pharmacokinetic (PK) and pharmacodynamic (PD) characteristics. These characteristics, which dictate the drug's interaction with biological systems, are subject to modulation by absorption, distribution, metabolism, and excretion (ADME) processes. A critical factor affecting these properties is the capability of a drug to bind to plasma proteins (PPs). Plasma protein binding (PPB) plays a pivotal role in determining a drug's ADME behavior, the relevant clinical outcomes, efficacy and toxicity. Understanding the molecular and physiological mechanisms underlying the binding of drugs, such as anticancer agents, to PPs is essential to gather substantial data for optimizing therapeutic outcomes.^1^ PPB is a fundamental attribute utilized to develop PK/PD relationships of the drug, predict drug–drug interactions (DDIs), and assess the toxicity of drug candidates. It is also crucial for predicting human PK using *in vitro* data.^2^ The small molecule drug binding to plasma and tissue proteins is very important in drug distribution from systemic circulation to extravascular spaces and impacts the hepatic and renal clearance.^3,4^ When drug molecules enter the systemic circulation, they exist either in free form or bound to PPs. Only free drug molecules are capable of passively diffusing through barriers into organ tissue, interacting with therapeutic targets to elicit pharmacological effects, also undergoing metabolism.^5,6^

As the most abundant protein in blood plasma, serum albumin functions primarily to carry other molecules, fulfills a central physiological role in drug transportation, distribution and metabolism. Human serum albumin (HSA) exhibits a strong binding affinity for ligand binding, encompassing a diverse array of drugs and metabolites, thereby functioning as a crucial mediator for a systemic transport for many endogenous and exogenous compounds.^7^ Drug-HSA binding studies are of considerable importance from a theoretical and practical standpoint, as they elucidate the mechanistic basis of drug bioavailability, and elimination. Furthermore, such studies shed light on the influence of diverse pathophysiological states and concurrent medications on drug delivery and therapeutic efficacy.^1^ These interactions can modulate several drug properties, such as increasing the solubility in plasma, mitigating toxicity, and prolonging the *in vivo* elimination half-life, thereby playing a dominant role in drug development and discovery.^8,9^

HSA is a single chain protein comprised of 585 amino acid residues and it is the most abundant protein in plasma, contributing approximately 60% of the total PP mass. The crystallographic structure of HSA has been established by conventional (classical) research to consist of three homologous α-helical domains (I, II, and III), which are composed of amino acid residues 1–195, 196–383, and 384–585, respectively. Two subdomains (IA, IB, IIA, IIB, IIIA, IIIB) make up each domain.^10–12^ Helical structure accounts for around 67% of HSA.^13^ Sudlow's sites I, II, and III, which are found in subdomains IIA, IIIA, and IB, respectively, are the three well-known ligand binding sites.^14–16^ HSA ability to bind multiple ligands makes it a versatile carrier for clinical treatment and drug delivery. Its biocompatibility, biodegradability, non-immunogenicity, and long half-life contribute to its effectiveness as a drug carrier. Additionally, HSA can accumulate in tumor tissues, making it a promising vehicle for targeted cancer therapies since tumor cells exhibit a higher uptake of HSA molecules compared to normal cells.^10^

Sunitinib (SU), a multitargeted TKI, inhibits receptors like PDGFR-β and VEGFR-2 (IC_50_ 10 nmol L^−1^ for both), and c-KIT, making it a standard treatment for highly vascularized renal cell carcinoma (RCC).^17–19^ Regretfully, several studies have documented resistance to SU therapy, highlighting the need to focus on other potential candidate treatments for RCC.^20–24^ This has spurred research into developing new compounds with improved PK profiles and anti-tumor activity. One promising approach is the synthesis of indolinone-*N*-substituted thiazolidinone/oxazolidinone hybrids, which could overcome resistance and enhance therapeutic overcome. A Sunitinib analog (SA) was synthesized and previously published by Dr Marwa A. Fouad *et al.* (Fig. 1),^25^ which has shown promising results. SA demonstrated excellent oral bioavailability, a broad range of cytotoxic activity against multiple tumor cell lines, and significant growth inhibition percent (GI%) in five renal cancer cell lines that were examined (A498, ACHN, CAKI-1, RXF393 and UO-31). SA also induced apoptosis and necrotic activity in renal cancer cells with different IC_50_ values ranging from 0.86 to 1.35 -folds, compared to SU. In addition, SA has moderate water solubility with no blood–brain barrier (BBB) permeability, meaning there won't be any expected CNS adverse effects.^25^

**Fig. 1:**
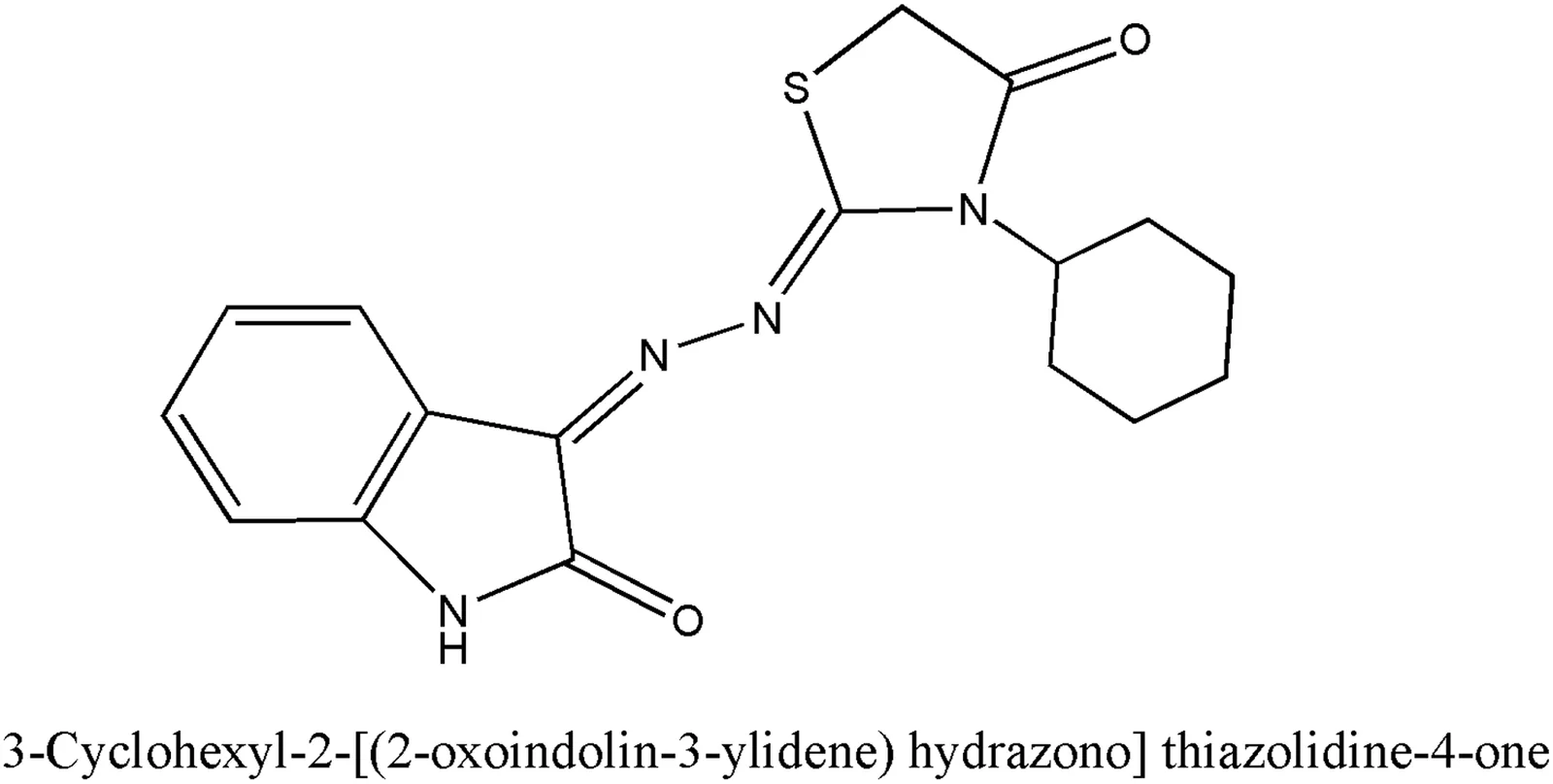
Chemical structure of SA.

Discerning the binding processes between this promising chemotherapeutic candidate, SA and HSA are essential to determine PKs and PDs that significantly influence the pharmacological properties of SA. The acquired knowledge holds significant value in guiding the design and refinement of novel SA. Therefore, the current study focused on characterizing the molecular interactions between SA and HSA. To address existing knowledge gaps in this domain, we have used both computational and experimental methods to study the mechanism of binding between SA and HSA. Through the implementation of suited multi-spectroscopic techniques, the thermodynamic properties and binding interaction mode between HSA and recently synthesized SA under physiologically simulated conditions (pH 7.4) were successfully determined. Furthermore, their binding location was examined using molecular docking simulation. This study is introduced to offer critical information regarding the storage and transport process of SA within human physiological system, as well as advancing the understanding of its pharmacological behavior performance and PK characteristics.

## 2. Experimental section

### 2.1 Chemicals and reagents

• Human serum albumin 20% solution (HSA) was purchased from Octapharma Pharmazeutika Produktionsges, mbH, Vienna, Austria, which was not purified before use. SA was synthesized in the lab according to the method in the reported literature.^25^ Digitoxin (99.05%), ibuprofen (100.4%), and phenylbutazone (99.54%) were supplied by EDA (Egyptian Drug Authority). Other chemicals were of suitable analytical reagent grade. Acetonitrile, liquid chromatography grade, was obtained from Merck (Germany). Disodium hydrogen phosphate (Na_2_HPO_4_) (≥99.0%) and dimethylsulfoxide were provided from Loba Chemie PVT, Ltd (India). Ethanol, methanol, and *ortho* phosphoric acid (≥85%) were obtained from Sigma-Aldrich, (Germany). Potassium dihydrogen phosphate (KH_2_PO_4_) (≥99.9%) was obtained from Scharlau (Spain). Potassium chloride (KCl) (≥99.9%) was provided by Piochem for laboratory chemicals, (Egypt). Sodium chloride; NaCl (≥99.9%) was purchased from Sigma-Aldrich (Germany). Ultrapure water, characterized by a resistivity exceeding 18.2 M Ω was generated using a Millipore Milli-Q^®^ UF-Plus purification system (Millipore, MA, USA).

• Isotonic PBS (Phosphate buffered Saline) (pH = 7.4) consisted of 137 mM of NaCl, 2.7 mM of KCl, 10 mM of Na_2_HPO_4_ and 1.8 mM of KH_2_PO_4_ (25 °C, pH = 7.4) by using *ortho* phosphoric acid.^1,26^ The pH measurements were made with a pH and conductivity meter (Jenway 3540; Cole-Parmer Ltd, Stone, Staffs, UK).

### 2.2. Instruments/apparatus

All fluorescence spectra were recorded on a Shimadzu spectrofluorometer RF-6000 combined with LabSolutions™ RF software (Japan) with 1 cm path-length quartz cell. The UV-1601 spectrophotometer (Shimadzu, Kyoto, Japan) was used to collect the UV-absorption spectra of all HSA solutions, both with and without SA, equipped with a pair of quartz cuvettes possessing optical path length of 1 cm. The Fourier Transform Infrared (FT-IR) spectra of HSA in the absence and presence of different concentrations of SA were recorded in the wavenumber range of 4000–400 cm^−1^, using Bruker Vertex 70v FTIR Spectrophotometer (Germany), combined with Opus software. The Molecular Operating Environment (MOE, 2024.06) software was used for computational work.

### 2.3. Procedures

#### 2.3.1. Preparations of stock and working solutions

The stock solution of SA (2 × 10^−3^ M = 2000 µmol L^−1^) (molecular weight 342 g mol^−1^) was prepared in a mixture of dimethyl sulfoxide (DMSO) and acetonitrile (3 : 1), respectively. The working solution (2 × 10^−4^ M = 200 µM) was prepared in isotonic PBS (pH 7.4) as simulated physiological conditions. To guarantee that organic solvents wouldn't alter the characteristics of HSA. In this study, a final solvent concentration of less than 1.5% was maintained in experimental solutions. The commercially purchased 20% HSA solution was diluted in isotonic PBS (pH = 7.4) to prepare the stock solution at a concentration of (1.0 × 10^−4^ M), and the working solution (1 × 10^−5^ M = 10 µM) was also prepared in isotonic PBS. Ethanol was used to prepare stock solutions of the site-specific markers (1000 µM each) for digitoxin and methanol for ibuprofen and phenylbutazone, respectively. While the working solutions were prepared by diluting the stock solutions with PBS to a 40 µM concentration. Every solution was stored at 4 °C in the dark.

#### 2.3.2. Fluorescence spectra measurements

For steady-state fluorescence (SSF), the excitation wavelength (*λ*_ex_) was set at 290 nm and the fluorescence emission spectra of solutions were collected in the range of 295–600 nm at three distinct temperatures (298, 303 and 310 K). The temperatures selected correspond to standard room temperature (298 K), a slightly elevated ambient temperature (303 K), and human physiological temperature (310 K). The slit widths of excitation/emission were fixed at 5/5 nm. The corresponding isotonic PBS (pH 7.4) solution was used as the correct background. The SA concentration varied from 7.5 to 30.0 µM (with 2.5 µM increments) with a constant HSA's concentration of 2 µM. The average of three scans was used to create each spectrum, and each experiment was repeated three times.

For synchronous fluorescence spectra (SFS), a fixed wavelength interval was maintained between the excitation and emission monochromator scans. The difference between the excitation wavelength (*λ*_ex_) and the emission wavelength (*λ*_em_) is represented by the Δ*λ*, which means (Δ*λ* = *λ*_em_ − *λ*_ex_). The values of Δ*λ* were set separately at 15 and 60 nm with a sampling interval of 1 nm.

For the 3D fluorescence (3DF) measurement, the excitation/emission wavelengths were set at 200–400/220–550 nm with bandwidths of 10/5 nm, respectively. The 3DF spectra were scanned with and without SA. The HSA concentration was kept constant (2 µM), whereas the concentrations of SA were varied as 5, 10, and 30 µM.

The data files were exported to a computing device and analyzed using OriginPro 8.5.1 software.

#### 2.3.3. UV- absorption measurements

At room temperature, measurements were made in the 200–600 nm spectral region under physiologically mimicked conditions (pH = 7.4). The concentration of SA was gradually raised from 2 to 30.0 µM while the concentration of HSA remained constant at 2 µM.

#### 2.3.4. FT-IR measurements

For all spectroscopic measurements, the spectra of HSA 2.5 µM in the presence of SA at concentrations from 0 to 10 µM (HSA–SA molar ratios of 1 : 0, 1 : 1, 1 : 2, and 1 : 4) were acquired with a total of 20 scans and a resolution of 4 cm^−1^. FT-IR spectroscopy can provide information about changes in the protein secondary structure upon ligand binding. The data processing was performed by digital curve-fitting and treated through self-deconvolution and Guassian peak fitting methodology^27^ using software OriginPro 8.5.1.

#### 2.3.5. Site marker competitive experiments

Three different site-selective markers, which are phenylbutazone (PB, as site I marker), ibuprofen (IBU, as site II marker), and digitoxin (DG, as site III marker) were used in the competitive displacement experiments. In this experiment, the concentration of SA (7.5–30.0 µM) was gradually added to the co-solution of HSA and marker, where HSA was fixed at (2 µM) and the molar ratio of site-marker/HSA was 2 : 1.

#### 2.3.6. Molecular docking study

The X-ray crystallographic structure of HSA co-crystallized with phenylbutazone, ibuprofen, and Mcl-1 Inhibitor (PDB ID: 2BXC, 2BXG, 5UJB),^28–30^ respectively were retrieved from the protein data bank (PDB) *via* (https://www.rcsb.org/pdb/home/home.do). In these downloaded HSA protein structures, PB molecules are bound to HSA site I, IBU is bound to HSA site II, and Mcl-1 Inhibitor is bound to HSA site III. Water molecules and ligands not involved in binding were eliminated after Chain B. Next, the MOE Quick Prep technique was used to prepare the protein structure using the default settings (structural correction, the addition of hydrogen atoms, the generation of conformational ensembles, neutralization of charged groups, creation of energetically advantageous conformers at physiologically realistic conditions (pH ∼7.4), and ligand optimization), then it was ready for molecular docking. Computational preparation of the ligand encompassed a series of optimization steps. The 3D structure of SA was constructed using Chem3D Ultra 8.0. The docking simulations were conducted with the following parameters: default energy minimization settings, the MMFF94 force field. The computational docking protocol employed dual scoring function, utilizing London dG as function 1 and (GBVI/WSA dG) as a secondary scoring metric.

#### 2.3.7. Suppression of inner filter effect

The fluorescence intensity is expected to diminish because of the presence of light absorbing species. The *I*_cor_/*I*_obs_ is proportional to exp(*A*) (*A* = (*A*_ex_ + *A*_em_)/2, *A* is the absorbance of determination system),^27^ which is defined as inner filter effect (IFE). The absorption and fluorescence tests were carried out to remove the IFE. Elimination of IFE is essential for improving the accuracy of fluorescence data. All fluorescence intensities in this study were adjusted for IFE throughout the subsequent eqn (1):

1 *F*_corr_ = *F*_obs_ × *e*^(*A*_ex_ + *A*_em_)/2^

In this case, the corrected and observed (experimentally determined) fluorescence intensities are denoted by *F*_corr_ and *F*_obs_, respectively, and *A*_ex_ and *A*_em_ refer to the total of the absorbances of SA and HSA at the emission and excitation wavelengths, respectively. As recommended by Lakowicz, the IFE adjustment was applied to each fluorescence intensity measurement.^31^

## 3. Results and discussion

### 3.1. Analysis of fluorescence quenching mechanism of HSA induced by SA

Proteins possess intrinsic fluorescence chiefly originated from the presence of aromatic amino acid moieties such as tryptophan (Trp), tyrosine (Tyr) and phenylalanine (Phe), which exert an imperative part in determining the fluorescence properties of macromolecules (like HSA and BSA). The inherent fluorescence of Phe is characterized by a negligible quantum yield, whereas the fluorescence of Tyr residues in protein can undergo substantial quenching through several mechanisms involving their local ionization mediated by neighboring carbonyl or amino groups present in the protein polypeptide's backbone.^27,32–34^ Nevertheless, a detectable Tyr contribution may be observed under experimental conditions and can be exploited in synchronous fluorescence measurements to monitor changes in the microenvironment of Tyr residues upon ligand binding. Among these residues, Trp is the predominant contributor of intrinsic fluorescence when excited at around 295 nm. HSA has a single Trp residue (Trp214) residing in Sudlow's site I of HSA,.^15,31^ Trp is highly sensitive to the changes in the protein's microenvironment and the polarity of the medium^31,35^ and has the strongest fluorescence intensity and is often employed as a fluorescent probe to examine the binding properties of compounds with protein. Variations in the emission spectra of Trp reflect the modification in the local environment of the indole ring.^36^

The quenching of protein fluorescence can generally be categorized into three mechanisms: the first one is static quenching, which arises from the formation of the ground-state non-fluorescent complex between the protein and quenchers. The second mode, termed dynamic or collisional quenching, arises from the collision of the fluorophore in protein with quenchers.^5,31^ While the third mechanism is combined (mixed) dynamic and static quenching, which is characterized by a non-linear upward curvature of the Stern–Volmer plot.^7,37^ A quenching mechanism is usually categorized according to how it behaves in relation to temperature. Consequently, temperature-controlled quenching experiments were performed to elucidate the specific mechanism of fluorescence quenching.^5,31,38^ A decrease in *K*_sv_ values with rising temperature indicates a static quenching mechanism, while a rise in *K*_sv_ values with temperature indicates the presence of dynamic quenching.^38^ Elevated temperatures promote molecular diffusion, thus enhancing the rate of dynamic quenching through augmented ligand–protein collisions, which manifests as elevated bimolecular quenching constants. Conversely, increased temperatures induce the detachment of labile ligand–protein complexes, leading to a reduction in complex stability and concomitant decline in static quenching phenomena.^31^

The fluorescence emission spectra of HSA obtained in the absence and presence of increasing concentrations of the SA were shown in (Fig. 2).

**Fig. 2:**
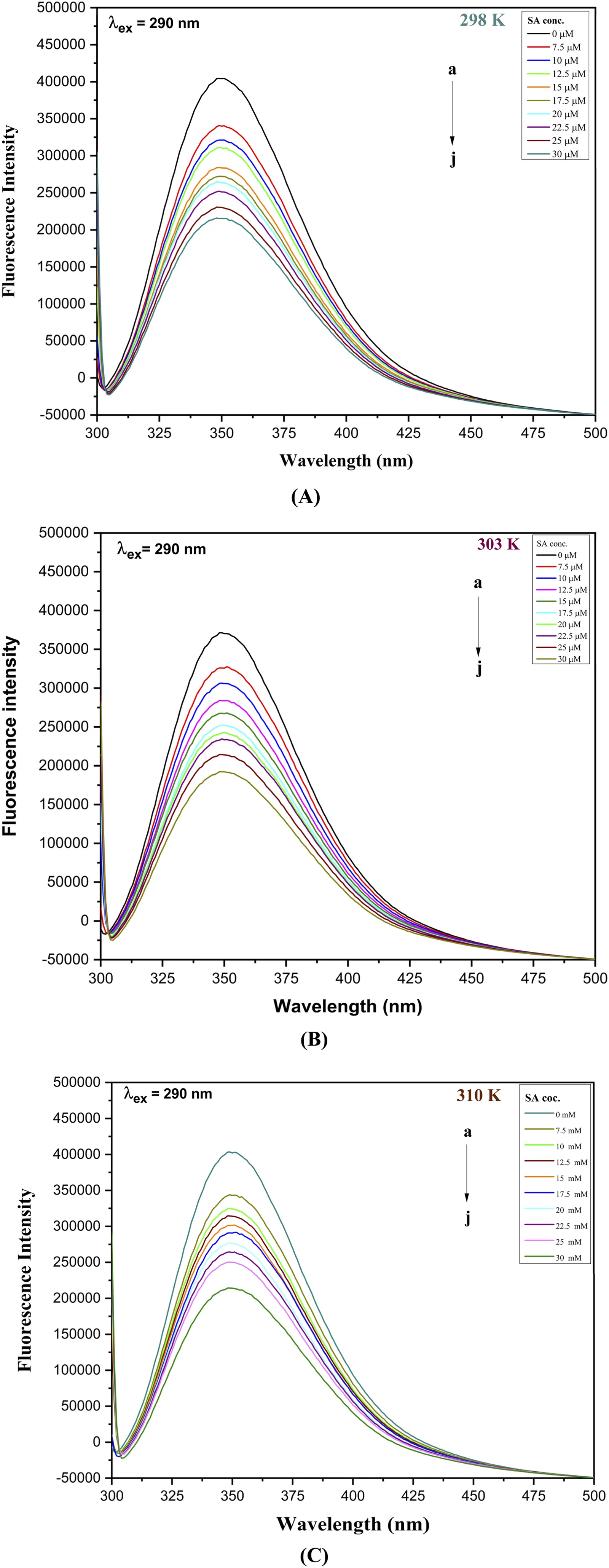
Fluorescence spectra of HSA solutions (2.00 µM) quenched by SA from a to j (0, 7.5, 10, 12.5, 15, 17.5, 20, 22.5, 25, 30 µM) at (A) 298 K, (B) 303 K, (C) 310 K.

Titrations were conducted at elevated temperatures, 298 K, 303 K, and 310 K, to elucidate the quenching mechanism underlying the interaction processes. Analysis of fluorescence data using classical eqn (2) yielded linear Stern–Volmer plots as illustrated in (Fig. 3), with the corresponding Stern–Volmer quenching constant (*K*_sv_) values summarized in (Table 1). HSA gives a strong emission peak centered at around 350 nm upon excitation at 290 nm. A consistent reduction in the fluorescence intensity of HSA was observed with increasing SA concentration. The results further revealed an inverse correlation between *K*_sv_ and temperature. Further, the results demonstrated that the calculated values of *K*_q_ considerably exceeded the maximum scattering collision quenching constant (2.0 × 10^10^ M^−1^ s^−1^).^39^ This suggested that complex formation, rather than dynamic collision, is the likely mechanism by which SA quenches the fluorescence of HSA.^40^

2 *F*_0_/*F* = 1 + *K*_sv_ [*Q*] = 1 + *K*_q_*τ*_0_ [*Q*]

In this context, *F*_0_ and *F* are SSF intensities measured in the absence and presence of the quencher molecule, respectively, and *K*_sv_ is the Stern–Volmer quenching constant. It makes the fluorophore accessible to the quencher. The concentration of quencher (SA) is represented by [*Q*], and the bimolecular quenching rate constant is represented by *K*_q_, and *τ*_0_ represents the average fluorescence lifetime of the fluorophore (HSA) when a quenching agent is absent.^41^

**Fig. 3:**
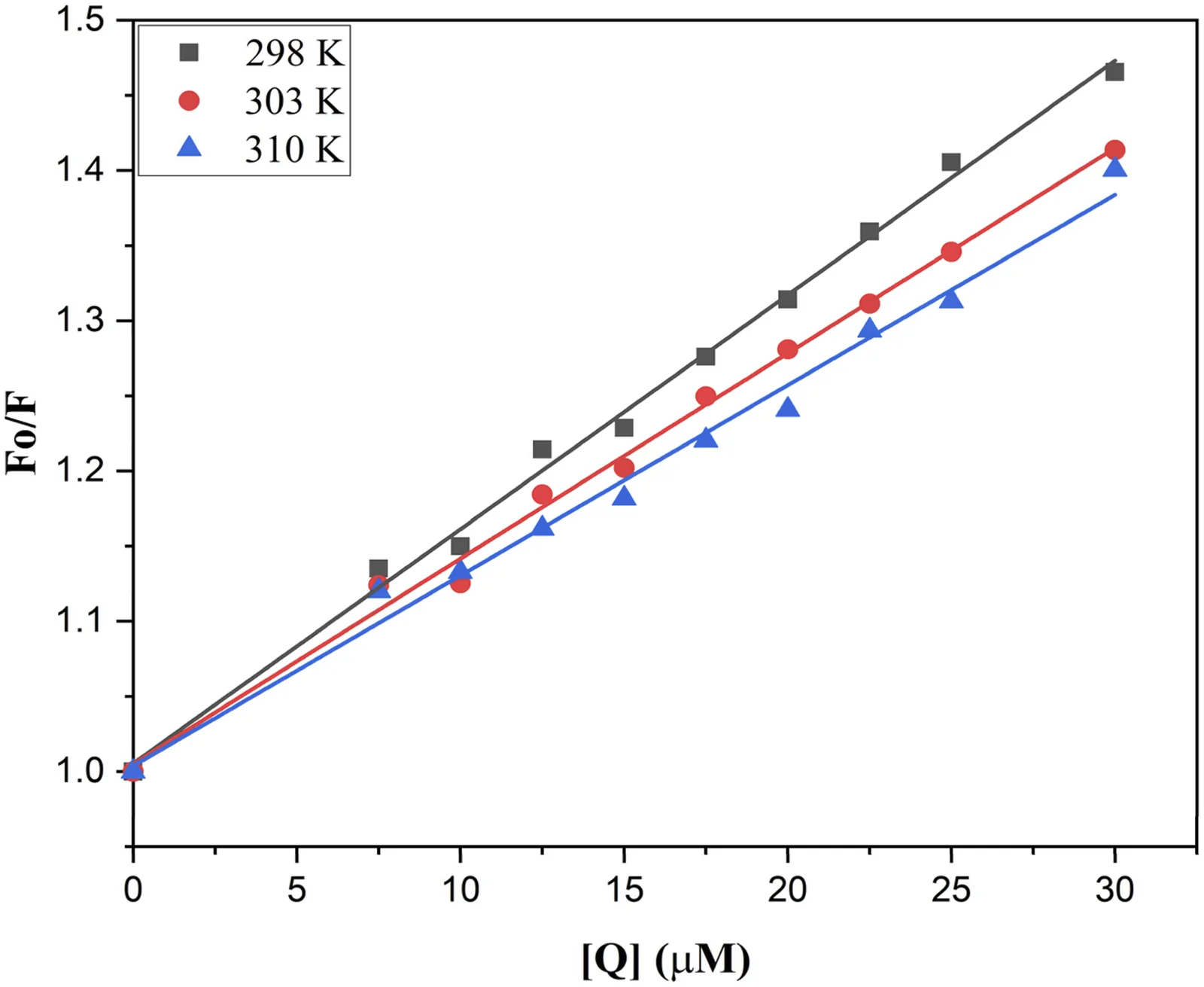
Stern–Volmer plot for the quenching of human serum albumin (2.0 µM) induced by SA at (298 K, 303 K, and 310 K).

**Table 1:** Stern–Volmer quenching constant, bimolecular quenching rate constants, binding constants, and number of binding sites for SA–HSA interaction system, studied at three different temperatures, (pH 7.4)

*T* (K)	Stern–Volmer equation			Modified Stern–Volmer equation		
	*K*_sv_ × 10^4^ (M^−1^)	*K*_q_ × 10^12^ (M^−1^ s^−1^)^a^	*r* ^2^ b	*K*_b_ × 10^4^ (M^−1^)	*n*	*r* ^2^ b
298	1.558 ± 0.002	2.442 ± 0.003	0.9949	3.257 ± 0.309	1.054 ± 0.005	0.9946
303	1.386 ± 0.019	2.173 ± 0.029	0.9950	1.743 ± 0.180	1.019 ± 0.008	0.9894
310	1.244 ± 0.023	1.949 ± 0.036	0.9888	0.882 ± 0.049	1.006 ± 0.003	0.9896

a *K*_q_ = *K*_sv_/*τ*_0_, *τ*_0_ ≈ 6.38 ns.

b *r* ^2^ is the coefficient of determination. (*K*_sv_: Stern–Volmer quenching constant; *K*_q_: bimolecular quenching constant; *n*: number of binding sites; *K*_b_: binding constant obtained).

The observed decline in the *K*_sv_ values (Table 1) with rising temperatures provides compelling evidence for the dominance of the static quenching mechanism in SA–HSA interaction, a process associated with the formation of stable complex between SA and HSA.

### 3.2. Binding constant and binding sites

Assuming independent binding of small molecules to a macromolecule, the presence of multiple binding sites (*n*) for the ligand on HSA can be inferred. The binding constant (*K*_b_) and the equilibrium between free and bound molecules are mathematically described through the double-logarithmic equation^42,43^ as shown in eqn (3) below:

3 log[(*F*_0_ − *F*)/*F*] = log *K*_b_ + *n* log[*Q*]

The value of binding constant (*K*_b_) was determined from the intercept of the linear regression analysis of log(*F*_0_ − *F*)/*F* plotted against log[*Q*] while that of the number of binding sites ‘*n*’ was obtained from its slope as illustrated in (Fig. 4).

**Fig. 4:**
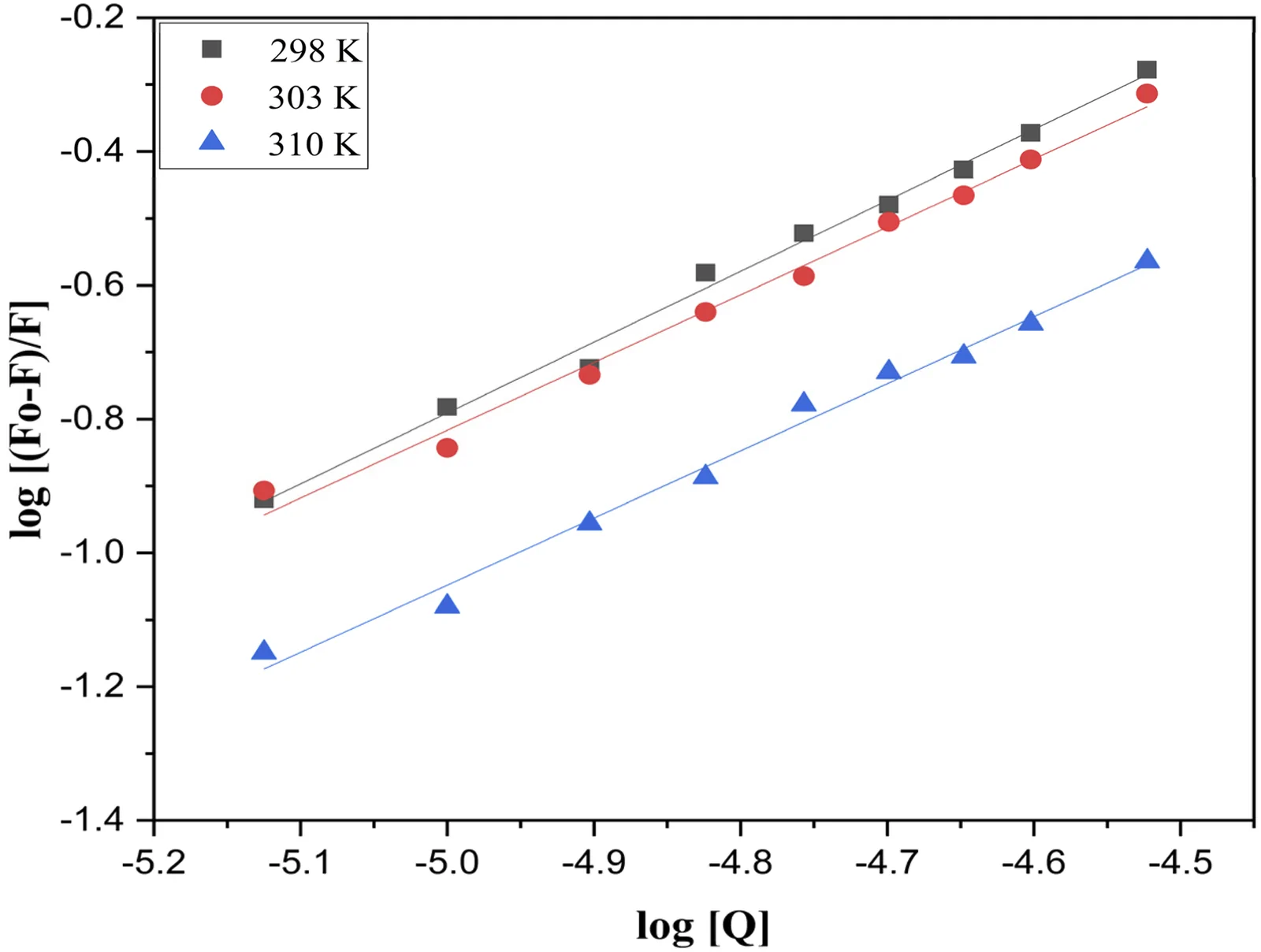
Best linear plot of log(*F*_0_ − *F*)/*F versus* log[SA] according to eqn (3) at 298, 303 and 310 K. Values of *K*_b_ and n of SA complex to HSA can be obtained from the slope and the intercepts, respectively.

The findings (Table 1) revealed that a single binding site for SA on HSA was expected to exist, and that one molecule of SA was bound to one molecule of HSA, as indicated by the values of (*n*) in the temperature range under study, which were close to unity.^44–46^ Moreover, the stability of the SA–HSA complex may have reduced with increased temperature, as seen by a drop in the *K*_b_ values with increasing temperature. The obtained values of log *K*_b_ (for 2 µM, HSA) were 4.505, 4.248, and 3.969 for 298, 303, and 310 K, respectively, suggesting that the binding affinity was moderate since a (log *K*_b_ 3.9–4.9) could be considered as weak to moderate strength of interaction.^47^ The magnitudes of obtained *K*_b_ values were at the level of 10^4^, indicating a moderately strong interaction^48^ between HSA and SA. Whereas a binding constant <10^3^ suggests low binding affinity.^49^ For a drug to be released from its bound form at the site of action and to ensure optimal delivery of it, a moderate association is preferred.^10^

### 3.3. Thermodynamic parameters and binding forces between SA and HSA

The established method to obtain information on the interaction forces from spectroscopic measurements is by evaluating the thermodynamic characteristics. The thermodynamic profile of the SA–HSA interaction was investigated by employing the van't Hoff equation shown below to analyze the binding data,^50^ thereby enabling the characterization of the operative intermolecular forces and the quantification of relevant thermodynamic parameters according to eqn (4):4
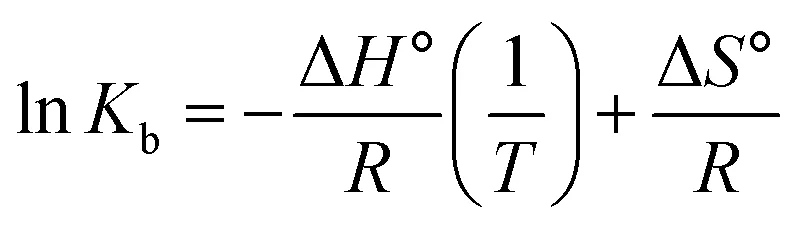
where *K*_b_ denotes the association constant at the corresponding *T*, *R* represents the gas constant (8.314 J mol^−1^·K^−1^),^51^ and *T* stands for the experimental temperature (in Kelvins).^52^ The primary variables utilized to reveal the binding mode are the thermodynamic parameters of enthalpy change (Δ*H*°), entropy change (Δ*S*°), and free energy change (Δ*G*°).^45^ The values of Δ*H*° and Δ*S*° were separately obtained from the slope and intercept of 1/*T versus* the ln *K*_b_ plot (Fig. 5) and the corresponding values are tabulated in (Table 2). Gibbs free energy change (Δ*G*°) can be calculated from the following relationship, eqn (5):

5 Δ*G*° = −*RT* ln *K*_b_ = −2.303 *RT* log *K*_b_

**Fig. 5:**
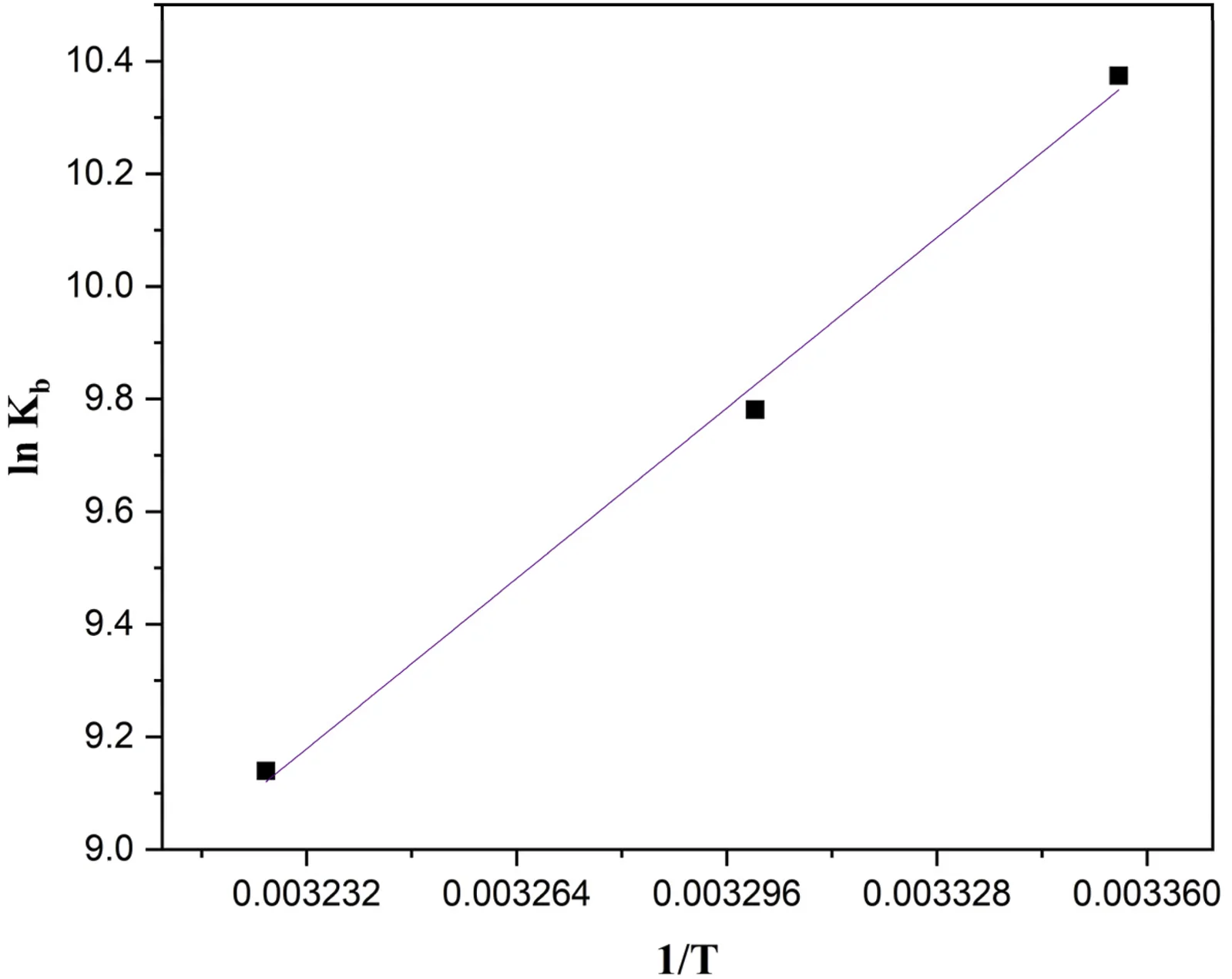
Plot of ln *K*_b_*versus* 1/*T*.

**Table 2:** Thermodynamic parameters for SA with HSA at different temperatures

*T* (K)	Δ*H*° (kJ mol^−1^)	Δ*S*° (J K^−1^ mol^−1^)	Δ*G*° (kJ mol^−1^)	*r* ^2^
298	−83.178 ± 2.605	−192.945 ± 5.668	−25.7464 ± 0.965	0.9964
303			−24.6033 ± 0.532	
310			−23.4165 ± 0.749	

According to Ross and Subramanian's conceptual model for association mechanisms,^45^ (1) when both Δ*H*° and Δ*S*° are negative, this indicates that the hydrogen bonding interaction and/or van der Waals force are the main force, (2) the preeminent interaction force is a hydrophobic interaction when both Δ*H*° and Δ*S*° are positive, and (3) the primary interaction force is the electrostatic force when Δ*H*° is close to zero and Δ*S*° is positive.^15,53^

Consequently, based on the previously outlined thermodynamic characteristics, as shown in the calculated results listed in (Table 2) the negative Δ*H*° and negative Δ*S*° values attained for the SA–HSA system disclosed the involvement of van der Waals force and/or hydrogen bonding interaction, which indicate that these forces play a major role in the SA–HSA binding interaction and contribute to the complex's stability and the negative value of Δ*H*° revealed the exothermic nature of the interaction.^54^ The hydrophobic cavity of site I was not significantly disrupted by the interaction of SA and HSA, as indicated by the negative value of Δ*S*°.^55^ Further, the negative Δ*G*° values for the system indicate that the interaction is spontaneous in nature.^56^ The large negative magnitude of these values indicates a highly thermodynamically favorable binding process, reflecting a strong binding affinity between the ligand and the protein.

### 3.4. Competitive binding experiments

It is commonly known that serum albumin possesses several noncovalent binding sites for compounds with low molecular weights. The most extensively researched out of all these sites are the subdomains IIA and IIIA, commonly referred to as Sudlow's sites I and II, respectively where Sudlow sites I and II are specific hydrophobic binding pockets located within subdomains IIA and IIIA.^57^ In addition, site III, which is in HSA subdomain IB, is the site that hemin and digitoxin prefer to bind to.^58^ Precise localization of drug-binding active sites on HSA is important since this information reveals the competitive mode of a drug. PB, IBU, and DG^49,59,60^ are recognized as site probes for Sudlow's sites I, II and III, respectively, according to the literature review.^10^ In contrast to site III in the HSA crystal structure, Sudlow's study indicates that site I and site II are more efficient binding sites. No discernible alterations are expected upon binding to the drug because site III has a lesser propensity for attracting molecules.^10,61^

The binding constants obtained for the ternary system (HSA -site marker-SA) in the presence of IBU (2.59 × 10^5^ M^−1^) were greatly different compared to either in the presence of PB (2.65 × 10^4^ M^−1^) or DG (3.61 × 10^4^ M^−1^) and of the binary system with no site marker (3.26 × 10^4^ M^−1^). In contrast, with PB and DG site markers present, there were only slight differences in the *K*_b_ values of HSA with SA plot (Fig. 6), and the corresponding values are tabulated in (Table 3). Thus, it can be deduced that the hydrophobic cavities of sub-domain IIIA (site II) of HSA may be the location of the binding site of SA. Also, some other TKIs such as bosutinib^46^ and Osimertinib^48^ were reported to be bound to serum albumin at site II in subdomain IIIA.

**Fig. 6:**
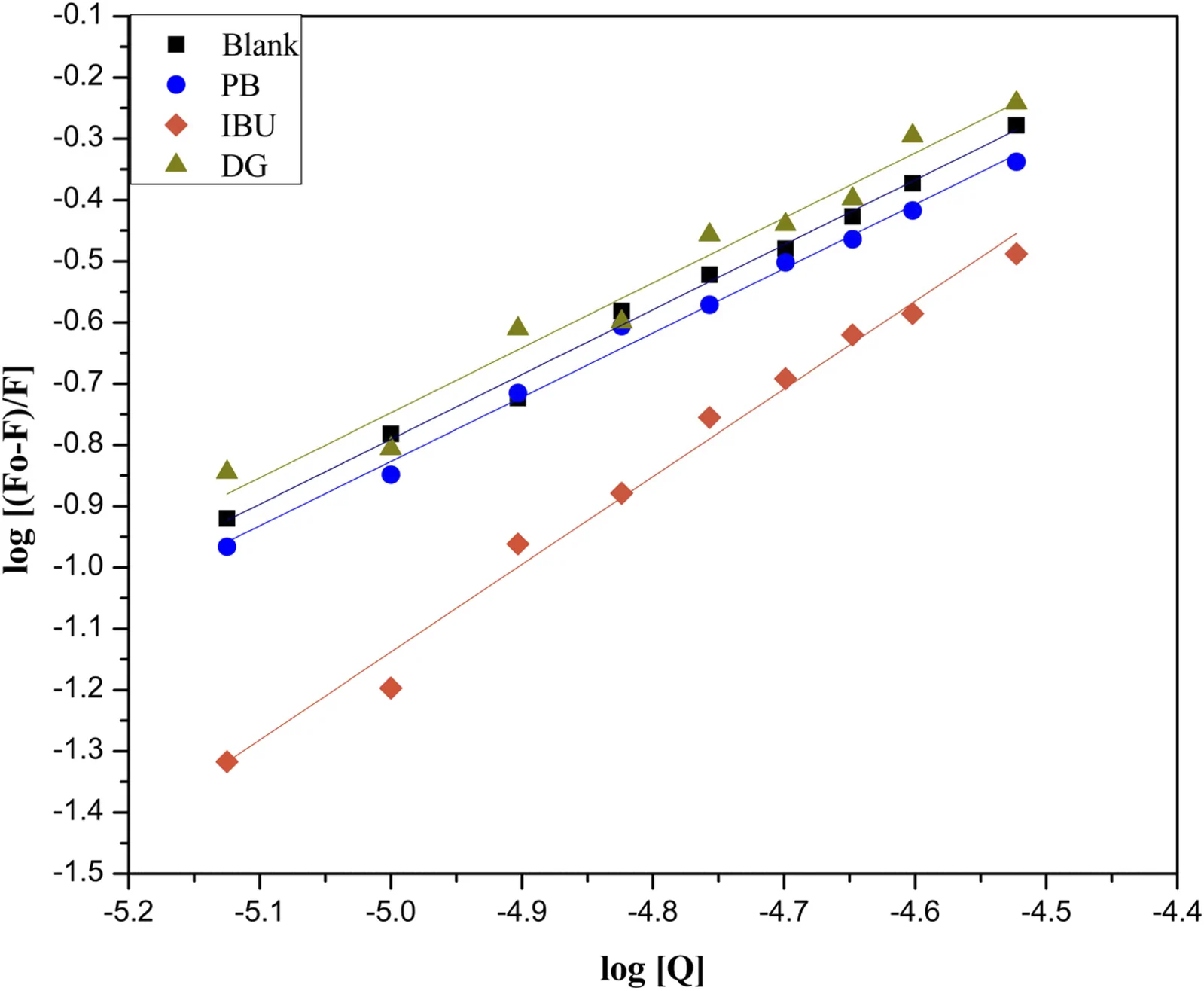
Modified Stern–Volmer plots for the binding of SA to HSA quenching by SA in absence and presence of site markers, *λ*_ex_ = 290 nm, [HSA] : [Competitive drug] = 1 : 2, 298 K, pH 7.4.

**Table 3:** Binding constants for SA binding into human serum albumin in the absence and presence of site-specific probes at room temperature

Site-specific probe	log *K*_b_	*K*_b_ × 10^−4^ (M^−1^)	*n*	*r* ^2^
HSA + SA (Blank)	4.512 ± 0.196	3.257 ± 0.309	1.0535 ± 0.005	0.9946
Phenylbutazone (PB)	4.424 ± 0.244	2.656 ± 0.080	1.0502 ± 0.007	0.9933
Ibuprofen (IBU)	5.414 ± 0.387	25.960 ± 0.615	1.3007 ± 0.003	0.9796
Digitoxin (DG)	4.558 ± 0.176	3.615 ± 0.116	1.0611 ± 0.009	0.9718

The binding constant obtained for the HSA–SA system increased markedly in the presence of IBU, from 3.257 ± 0.309 × 10^4^ M^−1^ to 25.960 ± 0.615 × 10^4^ M^−1^. Therefore, the observed increase cannot be explained by a simple competitive displacement mechanism. Instead, it may reflect an allosteric or conformational effect induced by IBU binding that enhances the apparent affinity of HSA toward SA. In contrast, the presence of PB resulted in a relatively small decrease in the apparent binding constant of SA (2.656 ± 0.080 × 10^4^ M^−1^), which may be consistent with some interaction between SA and the site I region, although the magnitude of this change does not provide conclusive evidence of direct competition. Taken together, these results support preferential interaction of SA with site II, while not completely excluding contributions from other binding regions.

### 3.5. Alteration in the protein's spectral characteristics upon SA addition (conformational changes)

#### 3.5.1 Synchronous fluorescence spectral analysis

Synchronous fluorescence spectroscopy (SFS) provides several advantages compared to SSF spectroscopy, including spectral simplicity and bandwidth reduction.^62^ Unlike SSF spectroscopy, SFS can offer specific information regarding the microenvironment in the vicinity surrounding distinct chromophores. Selective acquisition of spectral information pertaining Tyr and Trp residues was achieved by configuring the scanning intervals Δ*λ* (Δ*λ* = *λ*_em_ − *λ*_ex_) at 15 and 60 nm, respectively. The variations in the polarity of the microenvironment surrounding Tyr or Trp residues are reflected in the alterations in the maximum emission wavelength.^53^ The synchronous fluorescence spectra of HSA with the incremental addition of SA are presented in (Fig. 7). From (Fig. 7), it can be ascertained that the increase of SA concentration leading to a decline in the fluorescence intensities of Tyr and Trp residues, which is consistent with the SSF data. The maximum emission peak of Trp residues showed a blue shift of 2 nm from 340 nm to 338 nm upon adding SA from 0 to 30 µM, indicating the microenvironmental polarity around the Trp residue changed from being hydrophilic to hydrophobic. While a small red shift in the maximum emission wavelength of Tyr residue was observed (∼1 nm) from 297 nm to 298 nm, indicating a slight increase in polarity surrounding Tyr residue.

**Fig. 7:**
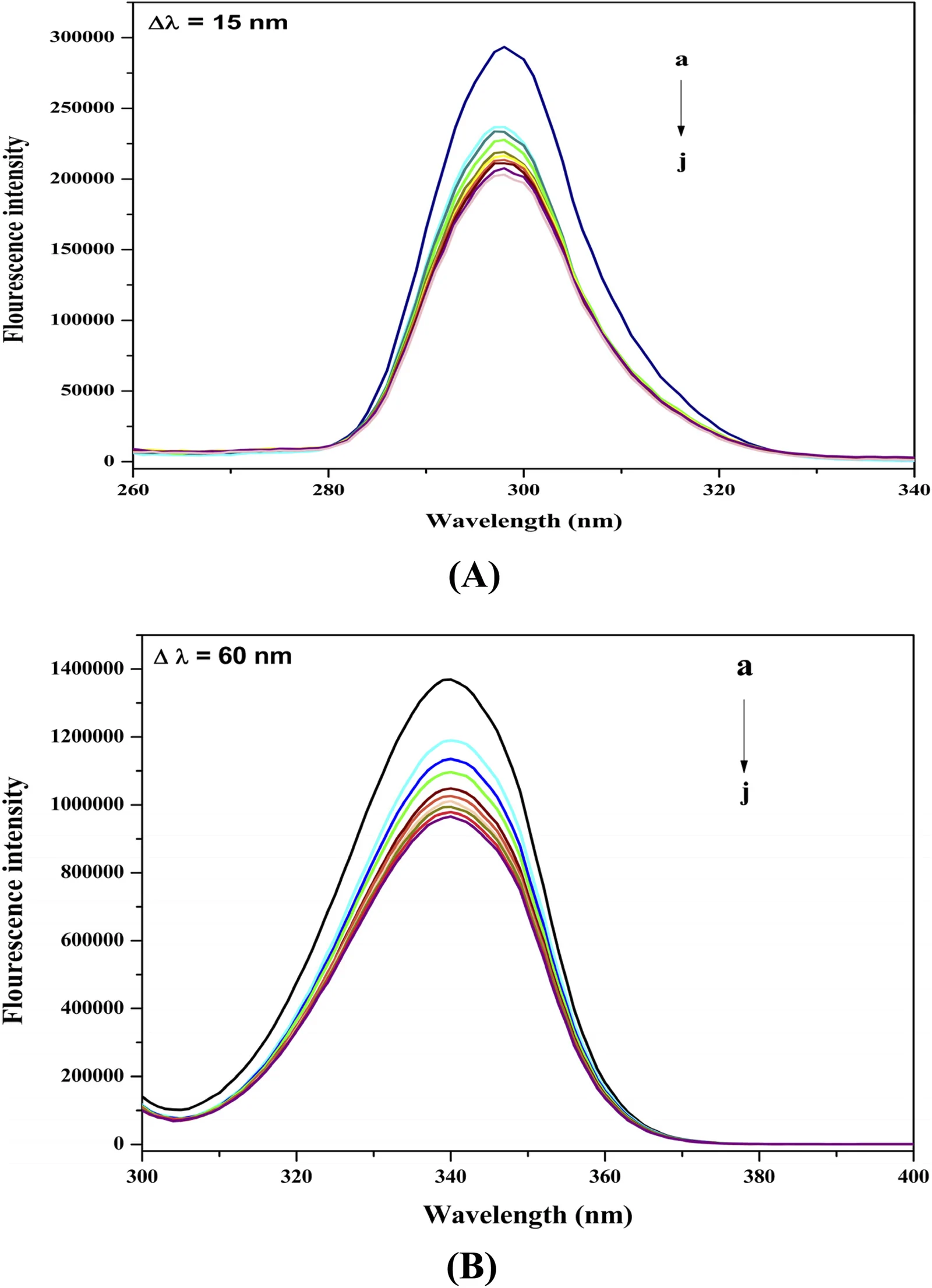
Synchronous fluorescence spectroscopy of HSA (2.0 µM) under simulated physiological conditions (pH = 7.4) at room temperature when Δ*λ* were set at 15 nm (A) and 60 nm (B), respectively. The concentrations of SA from a to j were 0, 7.5, 10, 12.5, 15, 17.5, 20, 22.5, 25 and 30 µM, respectively.

#### 3.5.2 UV- absorption spectral analysis

UV absorption spectroscopy is widely applied for investigating protein–ligand interaction. It can be used to distinguish the quenching mechanism. In case of static quenching mechanism, the formation of ground-state complex between the serum albumin and the ligand results in alterations in the absorption spectra with changing ligand concentration. Oppositely, in the case of dynamic quenching process, the excited state of fluorophore was only involved, leaving the absorption spectra of biomolecules unchanged with altering the concentration of ligand.^49^

UV absorption spectroscopy was used to investigate changes in the absorption characteristics of HSA upon interaction with SA. The UV absorption spectra of HSA in the absence and presence of increasing concentrations of SA were recorded in the wavelength range of 200–600 nm under simulated physiological conditions (pH 7.4), Fig. 8.

**Fig. 8:**
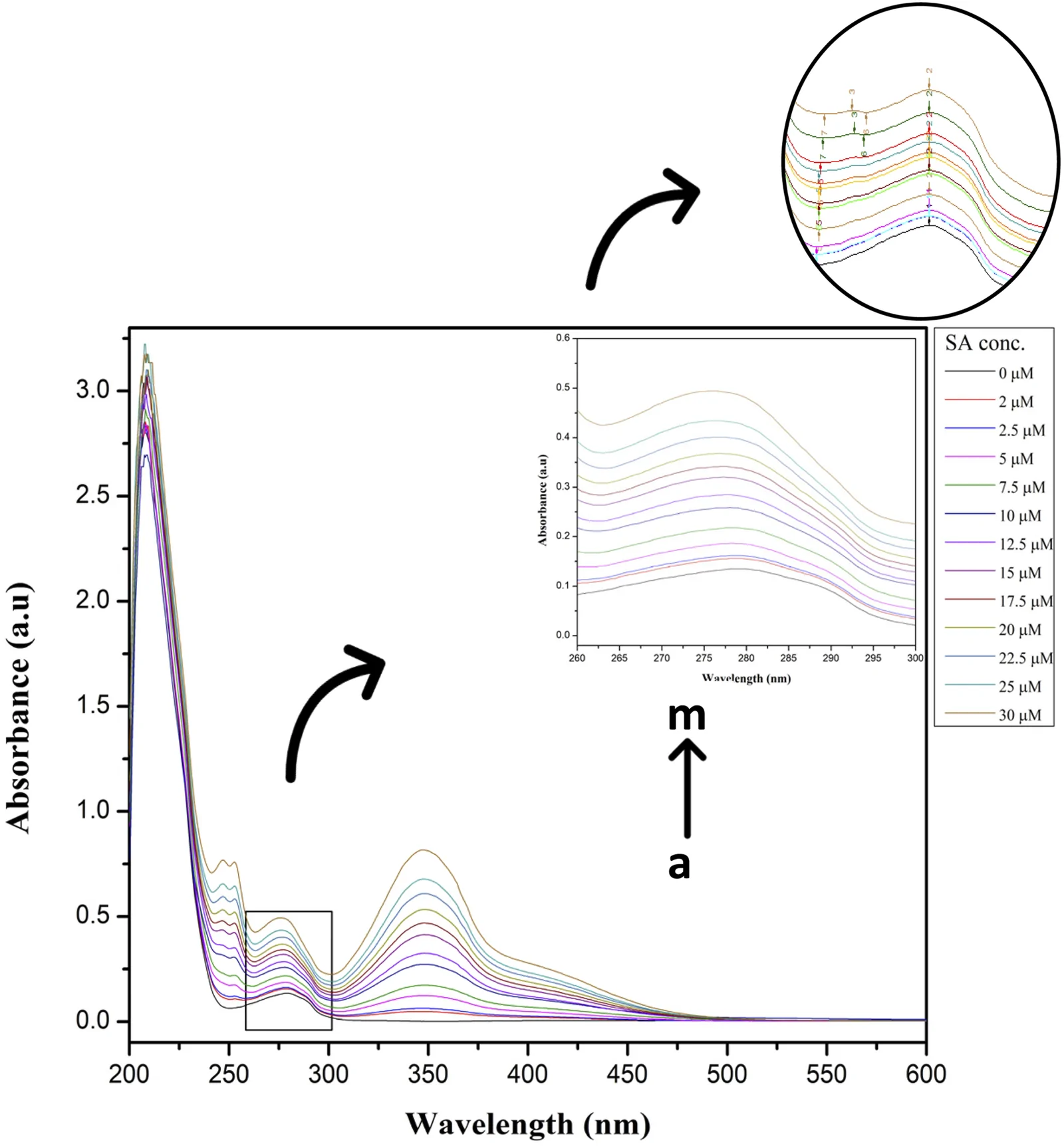
UV spectra of human serum albumin solutions (2.0 µM) in the presence of SA concentrations from a to m (0, 2, 2.5, 5, 7.5, 10, 12.5, 15, 17.5, 20, 22.5, 25, 30 µM) at room temperature.

HSA exhibited a strong absorption band in the low-wavelength region around 207 nm, mainly associated with the peptide backbone, and a weaker absorption band near 280 nm arising predominantly from the aromatic amino acid residues, including Trp, Tyr, and Phe.^63,64^ Upon addition of increasing concentrations of SA, changes in the overall absorption profile of the HSA solution were observed. However, because SA itself contributes to the UV absorption in this spectral region, the observed changes in the HSA–SA spectra cannot be attributed exclusively to changes in HSA. In addition, the high absorbance in the vicinity of 207 nm makes the assignment of a small wavelength shift in this region unreliable.

Accordingly, the UV absorption data were interpreted cautiously as indicating changes in the absorption characteristics of the HSA–SA system upon ligand addition, while the determination of the quenching mechanism was based primarily on the temperature-dependent fluorescence results. The decrease in the Stern–Volmer quenching constant with increasing temperature, together with the calculated bimolecular quenching constants exceeding the diffusion-controlled limit, provided the principal evidence supporting a predominantly static quenching mechanism.^63,65^ These findings show that the enhanced polarity (decreased hydrophobicity) surrounding the protein residue caused the peptide strands to expand more.^63,66^ Concomitantly, this behavior of UV data implies SA induced conformational remodeling of the HSA structure.

#### 3.5.3 Three-dimensional fluorescence spectral investigation

Nowadays, three-dimensional fluorescence (3DF) spectroscopy has emerged as a powerful and widely utilized technique for investigating conformational changes in proteins. In this method, three parameters are taken as outputs which are, *λ*_ex_, *λ*_em_ and fluorescence intensity, hence, this enhancing the precision and reliability of the results. 3DF spectroscopy is particularly effective for analyzing and portraying the microenvironmental perturbations proximate to aromatic fluorophores, such as Trp and Tyr, induced by ligand binding.^64,67^ The 3DF spectra and relevant contour maps of HSA–SA complex mixture in molar ratios 1 : 0, 1 : 5 and 1 : 15 are depicted in (Fig. 9(A–F)), respectively.

**Fig. 9:**
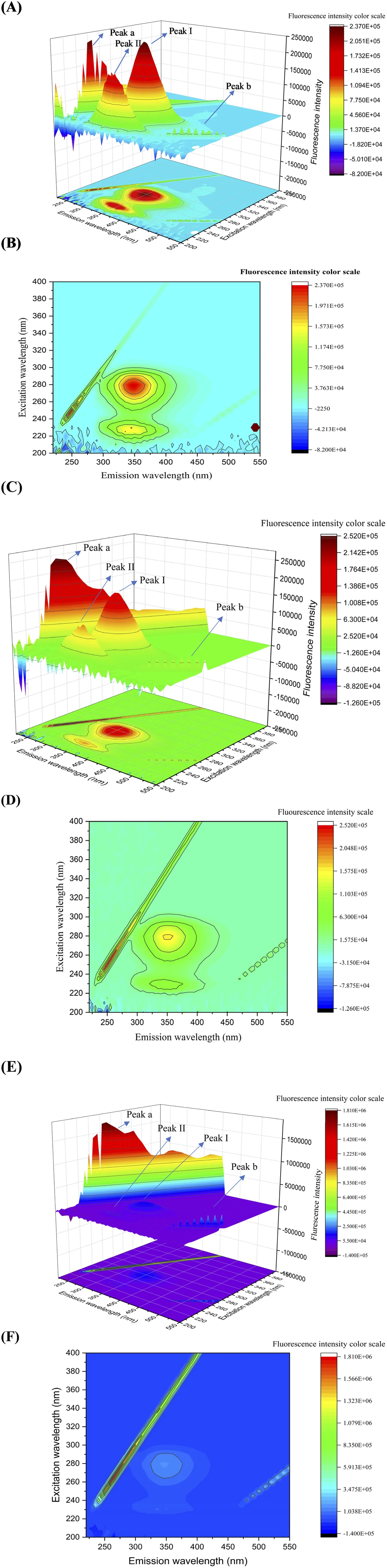
3D Fluorescence spectra of HSA in absence (A) or presence of SA. [HSA] = 2 µM, [SA] = 10 µM (C) and 30 µM (E), with their respective contour plots (B, D and F) and respectively.

Fig. 9(A–F) illustrates that four peaks were identified for HSA–SA system. Peak ‘I’, observed at an excitation wavelength of approximately 285 nm, is attributed to fluorescence emission peak due to Trp and Tyr residues; peak ‘II’, observed upon excitation at ∼225 nm, also originates from aromatic moieties Trp/Tyr emission under far-UV excitation, which can be excited through higher electronic states and subsequently emit from their lowest excited states rather than from the polypeptide backbone chain. The polypeptide backbone is considered non-fluorescent under these conditions.^68^ In addition, peak ‘a’ is the Rayleigh scattering peak (*λ*_em_ = *λ*_ex_); peak ‘b’ is the 2nd order scattering peak (2*λ*_em_ = *λ*_ex_).^64,69,70^ Peak ‘I’ had *λ*_ex_ = 285 nm and *λ*_em_ = 350 nm and peak ‘II’ had *λ*_ex_ = 225 nm and *λ*_em_ = 350 nm. In the present study, the fluorescence intensity of peak ‘a’ increased noticeably with the addition of SA, which might correspond to increased scattering intensity.^71^ (Table 4) shows that the fluorescence intensity of peak I and peak II of HSA decreased gradually as the concentration of SA increased along with a blue shift (5 nm and 10 nm), respectively. The decrease in fluorescence intensities of peak I and II following the addition of SA suggests changes in the local environments of aromatic residues of HSA upon ligand binding. These changes may reflect alterations in microenvironment of the intrinsic fluorophore residues (Trp and Tyr) associated with interaction between SA and HSA. However, changes in fluorescence intensity alone do not establish protein unfolding or specific conformational changes in the polypeptide backbone.^68^

**Table 4:** The difference 3D spectral data of HSA with the addition of different concentrations of SA

Sunitinib analog concentration (µM)	Peak type	Peak position *λ*_ex_/*λ*_em_ (nm nm^−1^)	Stokes shift, Δ*λ* (nm)	Fluorescence intensity
0	I	285/350	65	211 500
	II	225/355	130	157 109.8
5	I	285/345	60	156 833
	II	225/355	130	99 030.8
10	I	285/345	60	143 292
	II	225/345	120	75 043.93
30	I	285/345	60	132 853.5
	II	225/345	120	21 845.19

#### 3.5.4 Influence of SA on the secondary structure of HSA by FT/IR spectral study

FT-IR spectral data emphasizing the conformational change of HSA and offer insightful information about the secondary structural content of proteins.^51^ Proteins exhibit two characteristic absorption bands (amide I and amide II) in their IR spectra.^72^ These are the prominent vibrational bands of the protein backbone. The amide I band, observed within 1700–1600 cm^−1^ spectral region, arises primarily from stretching vibrations of the carbonyl (C=O) bonds in the peptide linkages with minor contributions from other vibrational modes. Whereas the C–N stretching coupled with the N–H bending mode causes the amide II band to form in the region of 1600–1500 cm^−1^.^63,72^ Compared to the amide II band, the amide I band is more susceptible to the changes in protein secondary structure. Thus, amide I band is more beneficial for secondary structure studies.^73^ The conformational change in HSA upon binding to SA was assessed by collecting FT-IR spectra of both free HSA and SA-bound HSA in isotonic PBS (pH = 7.4) and were recorded as shown in (Fig. 10). The FT/IR spectra in (Fig. 10) showed HSA had a remarkable change in the range of 1800–1400 cm^−1^ after binding to SA. Especially, the peak position of amide I bands exhibited a shift toward lower wavenumber. Both bands' positions altered after treatment with SA. The overall FT-IR spectra showed a shift of the band from approximately 1639.197 to 1633.411 cm^−1^ upon SA binding. Further curve-fitting analysis revealed changes in three fitted components, with peak positions shifting from 1682.587 to 1671.017 cm^−1^, from 1639.197 to 1633.411 cm^−1^, and from 1599.699 to 1579.414 cm^−1^.

**Fig. 10:**
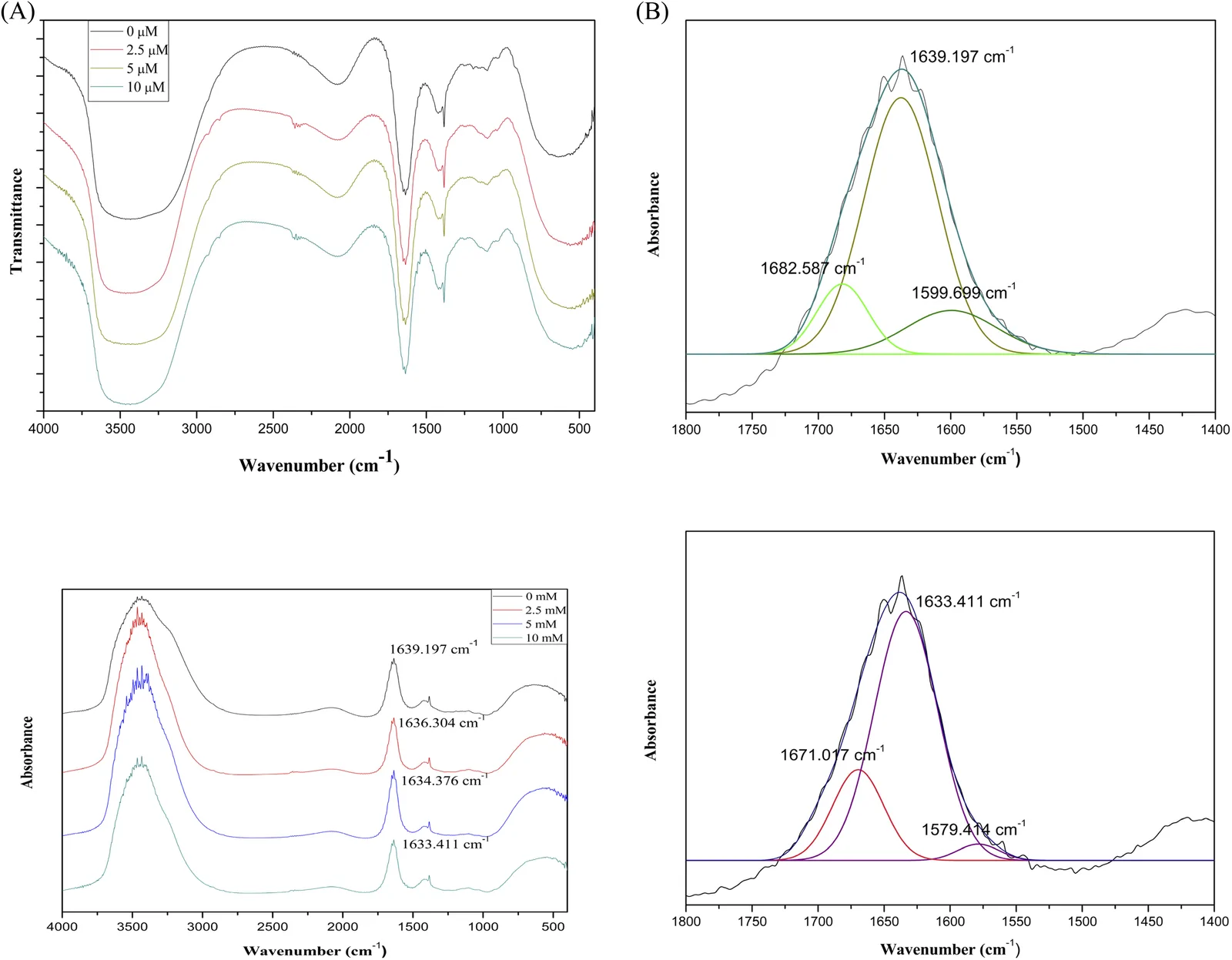
(A): FT-IR spectra of HSA in the absence and presence of SA. (B) The curve-fit of free HSA (Top) and HSA–SA (Bottom) at molar ratio of (1 : 4) in the amide I region from 1700 cm^−1^ to 1600 cm^−1^ and amide II region from 1600 cm^−1^ to 1500 cm^−1^ at room temperature.

The amide I and II bands were shifted from 1682.587 cm^−1^ to 1671.017 cm^−1^, and from 1599.699 cm^−1^ to 1579.414 cm^−1^, respectively upon interaction with SA. This shift implied that the protein's secondary structure had changed because of the HSA interaction with the SA.

### 3.6. Förster resonance energy transfer (FRET) between SA and HSA

Protein–ligand interactions can facilitate non-radiative energy transfer from HSA (donor) to SA (acceptor) can be analyzed by FRET. For this energy transfer to take place multiple conditions must be attained, that are: (1) the spectral overlap between the UV spectra of SA and SSF spectra of HSA, (2) the appropriate orientation between chromophores of the donor and acceptor molecules during the interaction, (3) the Förster distance between the donor and acceptor is typically in the range of 2–8 nm.^36,74,75^ The values of energy transfer efficiency (*E*) between the donor and acceptor, and the intermolecular distance (*r*) were quantified using mathematical relationships, as presented in the eqn (6) and (7):6
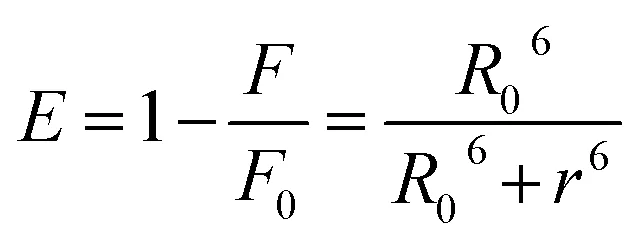
where *R*_0_ denotes the Förster distance at *E* = 50%, and *r* refers to the distance between donor and acceptor molecule. To calculate *R*_0_, eqn (7) can be used:

7 *R*_0_^6^ = 8.8 × 10^−25^*k*^2^*n*^−4^*∅J*

Here, *k*^2^ represents the dipole spatial orientation factor, *n* denotes the average refractive index of the medium, *∅* corresponds to the fluorescence quantum yield of the donor, and *J* signifies the spectral integral overlap between the emission spectrum of the HSA and the absorbance spectrum of SA.^7,76^ In this work: *k*^2^ = 2/3, *n* = 1.336, and *∅* = 0.118 are employed for the random orientation. *J* can be calculated from eqn (8), given below:8
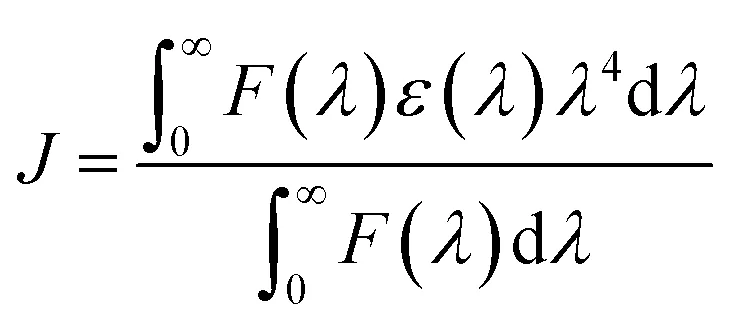
*F*(*λ*) is the normalized fluorescence intensity of the donor (HSA) of wavelength *λ* and *ε*(*λ*) is the molar extinction coefficient of the acceptor (SA) in M^−1^ cm^−1^. Additionally, from (Fig. 11), the obtained *J* value is 3.16 × 10^−13^ cm^3^ M^−1^. For SA and HSA interaction, *E* = 21.53%, *R*_0_ = 4.36 nm, and *r* = 5.41 nm. The results revealed that *r* value is lower than the maximum intermolecular distance (8 nm), larger than *R*_0_ value, and between the range 0.5 *R*_0_ < *r* < 1.5 *R*_0_, demonstrating the high possibility of a non-radiative energy transfer during SA binding with HSA and the static quenching mechanism is predominantly involved for the interaction between HSA and SA.^77^

**Fig. 11:**
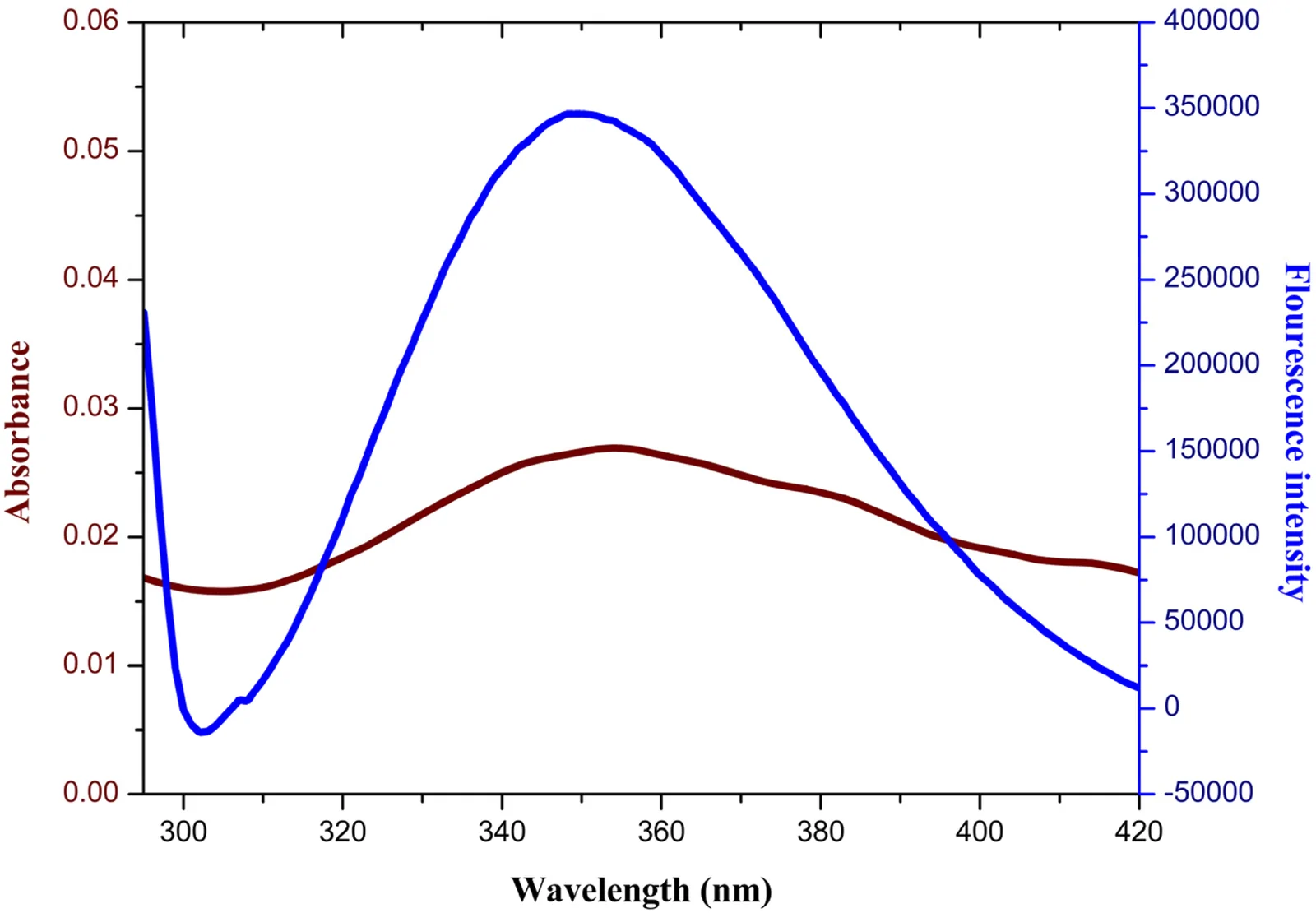
Spectral overlap between HSA fluorescence emission spectrum (2 µM) and the absorption spectrum of SA (7.5 µM) at 298 K.

### 3.7. Molecular docking of SA and HSA

The interacting residues and their positions were determined using the molecular docking model, which made use of the crystal structure of HSA. The most significant ligand binding site in HSA is located in domain II, according to molecular docking research (Ser342, Val344, Arg348) and domain III (Pro384, Leu387, Ile388, Asn391, Tyr411, Val415, Leu457, Phe488).^36^

The docking setup was first validated by carrying out self-docking of the relevant co-crystallized ligand for each binding site. The self-docking validation replicated the co-crystallized ligand experimental binding pattern in each binding site indicating that the used docking protocol is appropriate for the intended molecular docking study, this was demonstrated by the small RMSD between the experimental co-crystallized poses and the docked poses, with a predicted docking energy score of −11.0936, −10.9066 and −16.9795 Kcal mol^−1^ in drug site I, II and III, respectively for each ligand in its selective binding site (Table 5). Shows the docking scores for the different ligands in HSA drug sites I, II, and III. As can be seen in (Table 5), the docking scores reflect the experimental preferentiality of the different ligands to their HSA drug sites.

**Table 5:** Docking energy scores (S) in kcal mol^−1^ for phenylbutazone, ibuprofen, digitoxin, and SA in HSA drug sites I, II, and HSA drug site III

Compound	Docking score site I (phenylbutazone)	Docking score site II (ibuprofen)	Docking score site III (digitoxin)
Phenylbutazone	−11.0936	—	—
Ibuprofen	—	−10.9066	—
Digitoxin	—	—	−16.1744
SA	−12.2429	−12.4400	−10.3246

Phenylbutazone (PB) is a selective drug site I ligand. Its docking score in drug site I is more negative than that in drug site II, −11.0936 *vs.* −7.9010 kcal mol^−1^, respectively, indicating its higher binding to drug site I. PB binding pattern in HSA drug site I shows a hydrogen bond with Try150, Arg222, and Arg257 amino acids. On the other hand, ibuprofen is a selective drug site II ligand. So, its docking score in drug site II is more negative than in drug site I, −10.9066 *vs.* −9.4737 kcal mol^−1^, respectively, indicating its stronger binding to drug site II. Site II of HSA has a lower volume than site I and it consists of one main polar patch formed by Arg410, Tyr411, and Lys414 and a single hydrophobic chamber.^47^

The Ibuprofen (IBU) binding pattern in HSA drug site II (which is also known as the indole-benzodiazepine site)^78^ suggests hydrogen bonding interactions. The carboxylate group of IBU is oriented towards a basic polar patch formed by Arg410, Lys414 and Ser489, allowing it to act as a hydrogen-bond acceptor. Hydrogen-bonding interactions involving the carboxylate group and the positively charged side chains of residues N-Lys414 and O-Ser489 were observed in some docking poses but not necessarily in all poses.^79^ Through hydrogen bonding, it interacts by its carboxylate moiety with the side chain hydroxyl group of Tyr411 and Arg410. In addition, it interacts by π–H interaction with Leu453 amino acid.

The docking conformation showed SA interacts in site II through the stronger H-bonding interactions, whereas only two H-bonds formed at site I (Fig. 12). Whereas the docking of SA on site II showed interactions with the important active site residues Arg410, Tyr411, Lys414, and Leu453 in drug site II (Fig. 13). The hydrophilic head of our ligand, SA, interacts with the polar patch of site II and is responsible for the H-bonding with that site. There were two hydrogen bond acceptor (HBA) sites on the SA, corresponding to the carbonyl oxygen of the oxindole moiety and thiazolidine-4-one group. These two favorable acceptor features corresponded to the HBA interaction in the basic side-chains regions of Arg410 and Lys414, respectively. The carbonyl oxygen of SA formed hydrogen bonds with active site residues Arg410 and Lys414 at the distance of 2.39 and 2.06 Å, respectively. In addition, at 1.97 Å, there is a H-bonding present with Tyr411's hydroxyl group (Fig. 14). The docked SA molecule projects its single phenyl ring into the near right-hand sub-chamber of the pocket. Also, the SA binding pattern in HSA drug site II shows one arene–H interaction by its indole moiety with Leu453 which was demonstrated in the binding of IBU. Asn391 and Phe403 are two examples of residues that are at receptor exposure and somewhat close to the ligand but still not at a binding distance.

**Fig. 12:**
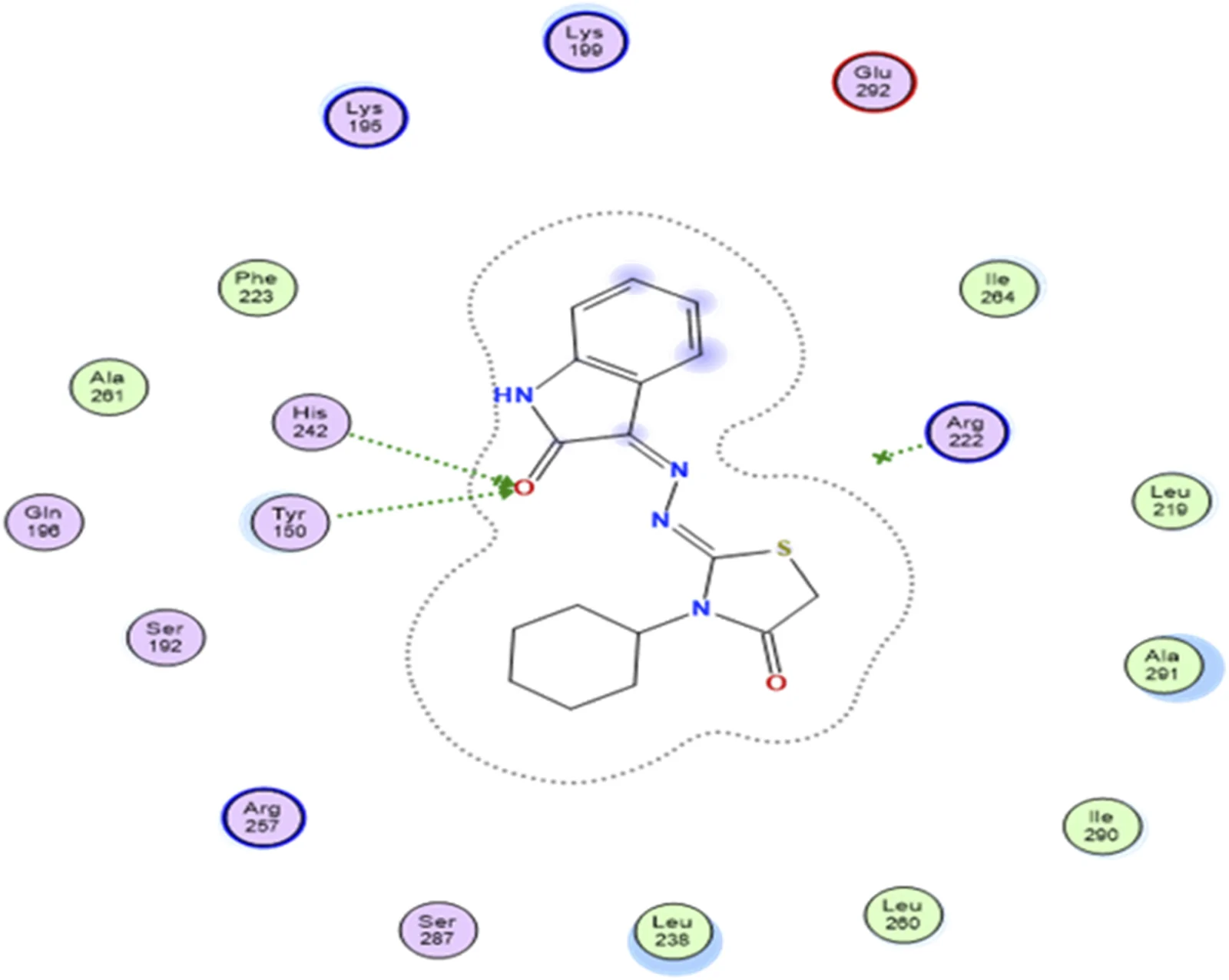
2D diagram of SA showing its docking pose interactions with the key amino acids in HSA drug site I.

**Fig. 13:**
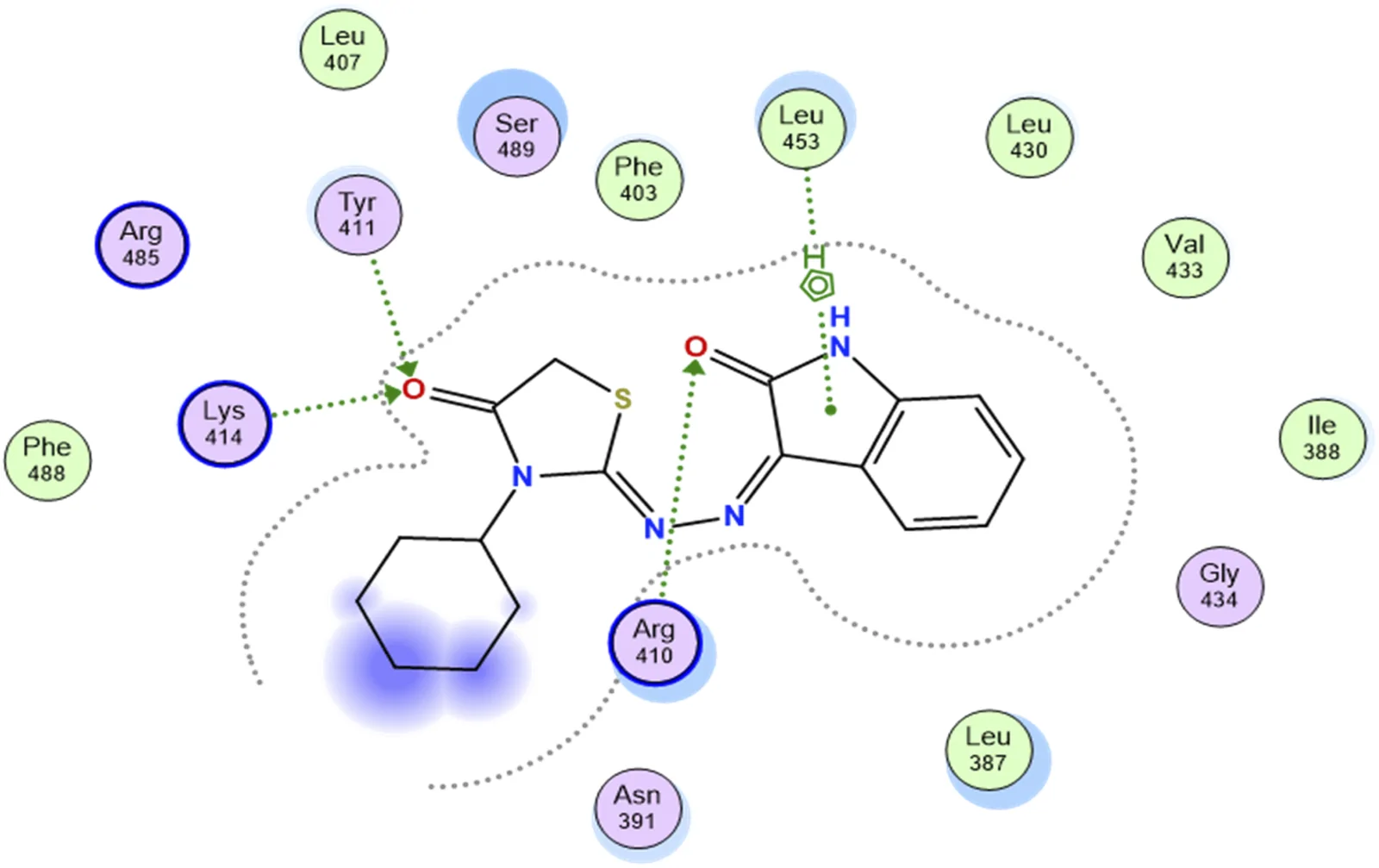
2D diagram of SA showing its docking pose interactions with the key amino acids in HSA drug site II.

**Fig. 14:**
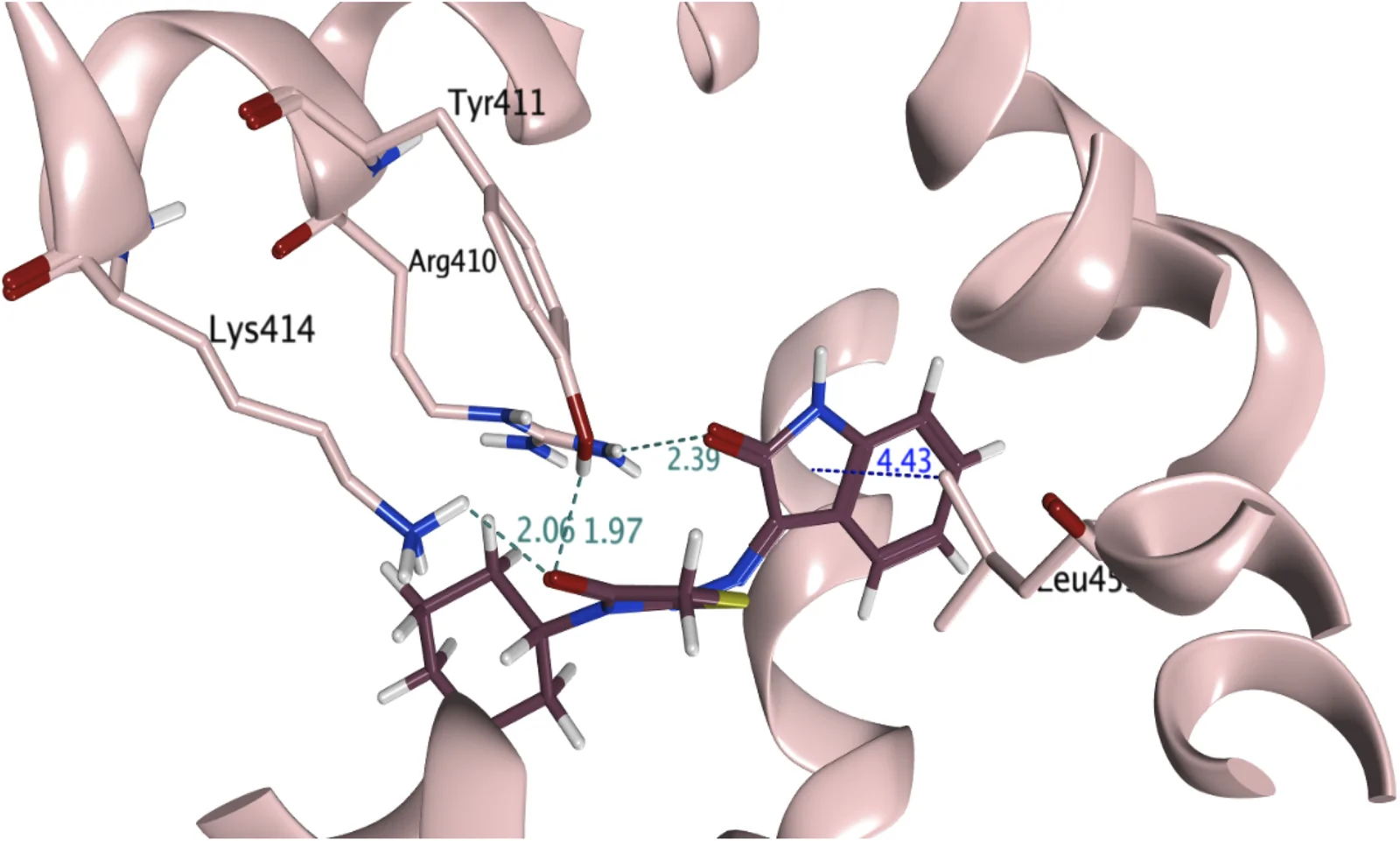
3D diagram of SA showing its docking pose interactions with the key amino acids in HSA drug site II.

It was proposed that ligands bonded to site III would generate polar interactions with Arg117 and Arg186.^47^ In the case of SA, these interactions were absent, as seen in (Fig. 15).

**Fig. 15:**
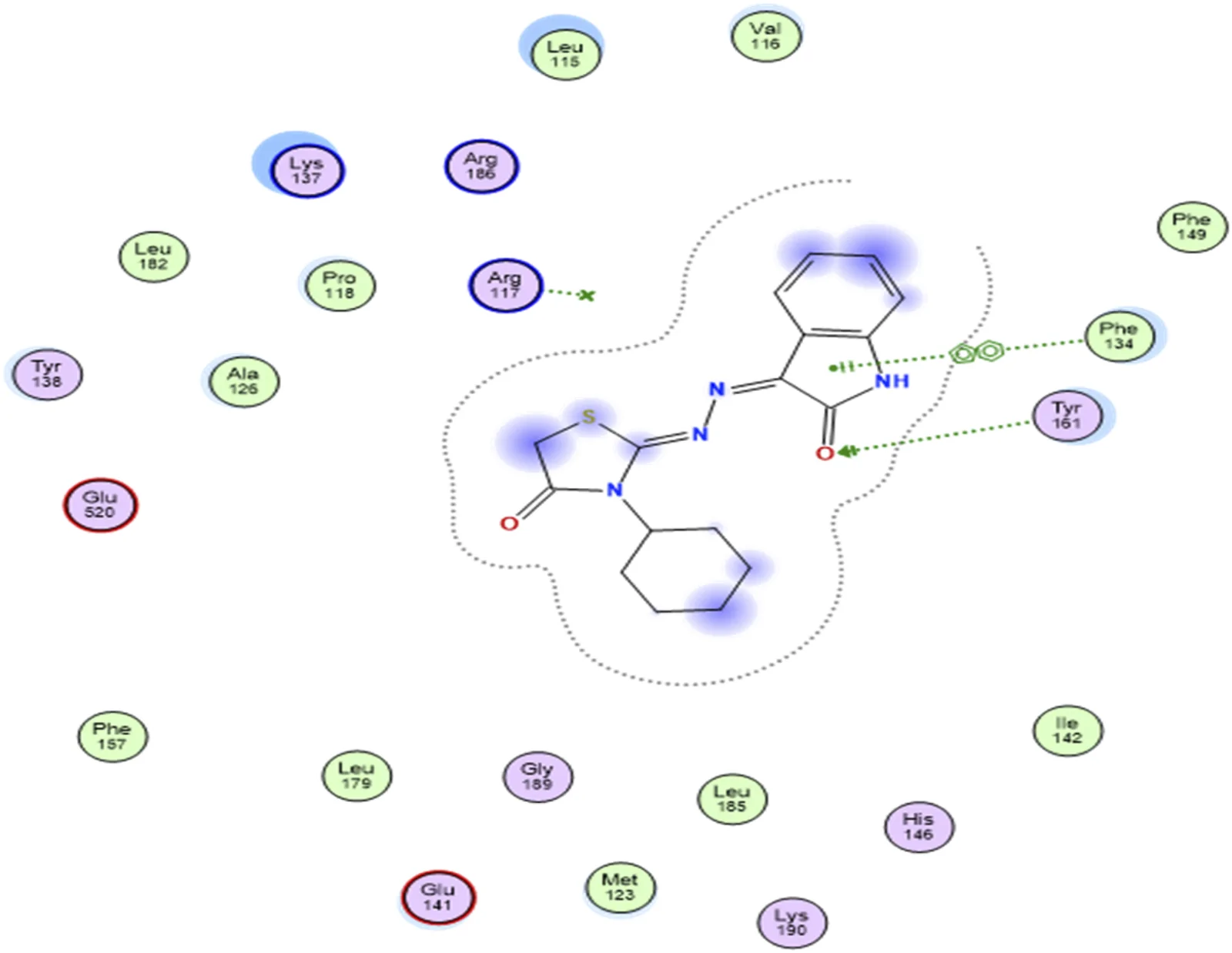
2D diagram of SA showing its docking pose interactions with the key amino acids in HSA drug site III.

SA has a high docking score (−12.4400) to site II, and strong binding. This indicates that the binding site is fully occupied, indicating strong binding. The hydrophobic nature of this interaction —in which the carboxylate group moiety interacts with charged amino acid residues in or near the hydrophobic cavity, while the aromatic moieties interact with Sudlow's site II of HSA—may be conferred by the presence of an aromatic ring in SA.

By comparing the interaction in the 3 sites, SA interacts in site II through the stronger H-bonding interactions, whereas, in site I and site III through a relatively weaker interaction, which may rationalize its preferential binding to site II.

## 4. Conclusion

Interaction between any drug molecule and HSA can lead to the formation of a stable protein–drug complex, which is a recognized determinant of drug pharmacokinetics. This study elucidated the binding interaction of a recently synthesized sunitinib analog (SA); a promising anticancer drug to the main ligand transporter protein in blood plasma, HSA. Molecular docking studies and multi-spectroscopic methods including fluorescence and UV absorption were used to investigate the binding mechanism of SA to HSA. Fluorescence spectroscopic analyses demonstrated that the fluorescence intensity of HSA was notably quenched by SA. The observed inverse relationship between the Stern–Volmer quenching constants (*K*_sv_) value and temperature pointed towards that SA induces a static quenching mechanism. An intermediate binding affinity stabilized the SA–HSA complex, as suggested by the binding constant (*K*_b_) value. In addition, competitive displacement experiments utilizing site-specific markers, including phenylbutazone, ibuprofen, and digitoxin, supported Sudlow's site II as the preferential binding region of SA, while a contribution from other binding regions, particularly Site I, cannot be completely excluded. This finding was further corroborated by molecular docking studies. The combined results of UV absorption, FT-IR, synchronous fluorescence, and 3D fluorescence spectral analysis collectively evidenced alterations in the microenvironment surrounding Trp and Tyr residues of HSA as well as conformational alteration in the protein backbone following the introduction of SA. The results and outcomes derived from this study would furnish pertinent information for explaining the binding process of SA-albumin *in vivo*. The conformational perturbations of the protein by SA offers critical insights into the biological pathway regulating drug delivery and associated processes within a cellular milieu.

## Author contributions

Sohaila A. Abbas: data curation, formal analysis, writing – original draft. Eman A. Mostafa: investigation, supervision, project administration, writing – review & editing. Alshaimaa M. Alian: supervision, review & editing. Ehab F. ElKady: supervision, project administration, writing – review & editing. Marwa A. Fouad: supervision, validation, conceptualization, project administration, writing – review & editing.

## Conflicts of interest

All authors have no conflicts of interest and no competing interests to declare.

## Supplementary Material

RA-OLF-D6RA07888E-s001

## Data Availability

All the data supporting this article has been included as part of the manuscript. Supplementary information (SI): spectral data of compound SA. See DOI: https://doi.org/10.1039/d6ra07888e.
