# Supplementary material for: Probing the binding dynamics of a sunitinib analog with human serum albumin: a combined spectroscopic and molecular docking approach
Source: RSC Adv. 2026 Nov 2. Online ahead of print. doi: 10.1039/d6ra07888e (PMC13725782; doi:10.1039/d6ra07888e)
Supplement: Supplementary file 1 [file RA-OLF-D6RA07888E-s001.pdf]

Supplementary Information (SI) for RSC Advances.
This journal is © The Royal Society of Chemistry

Spectral Data of Compound SA

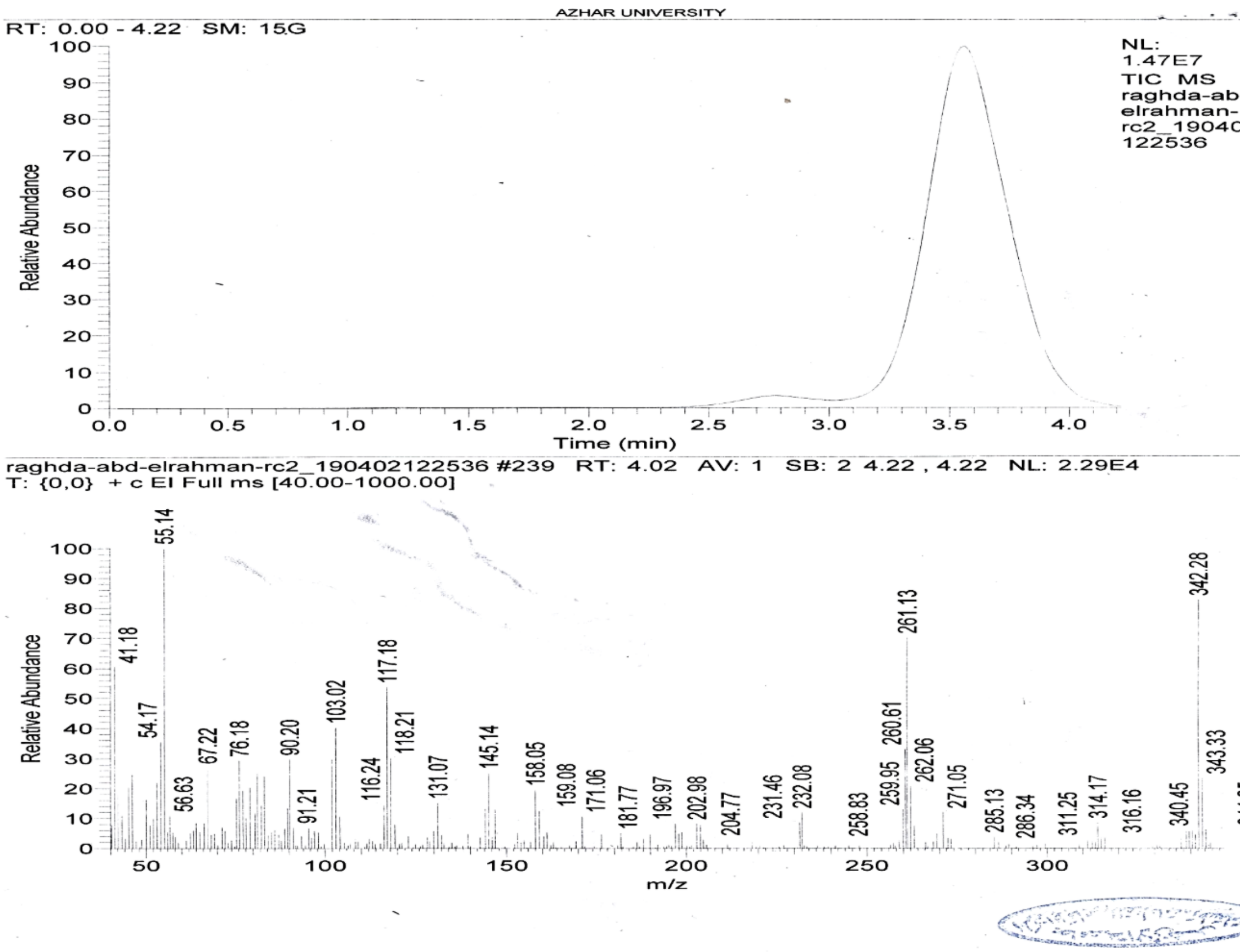

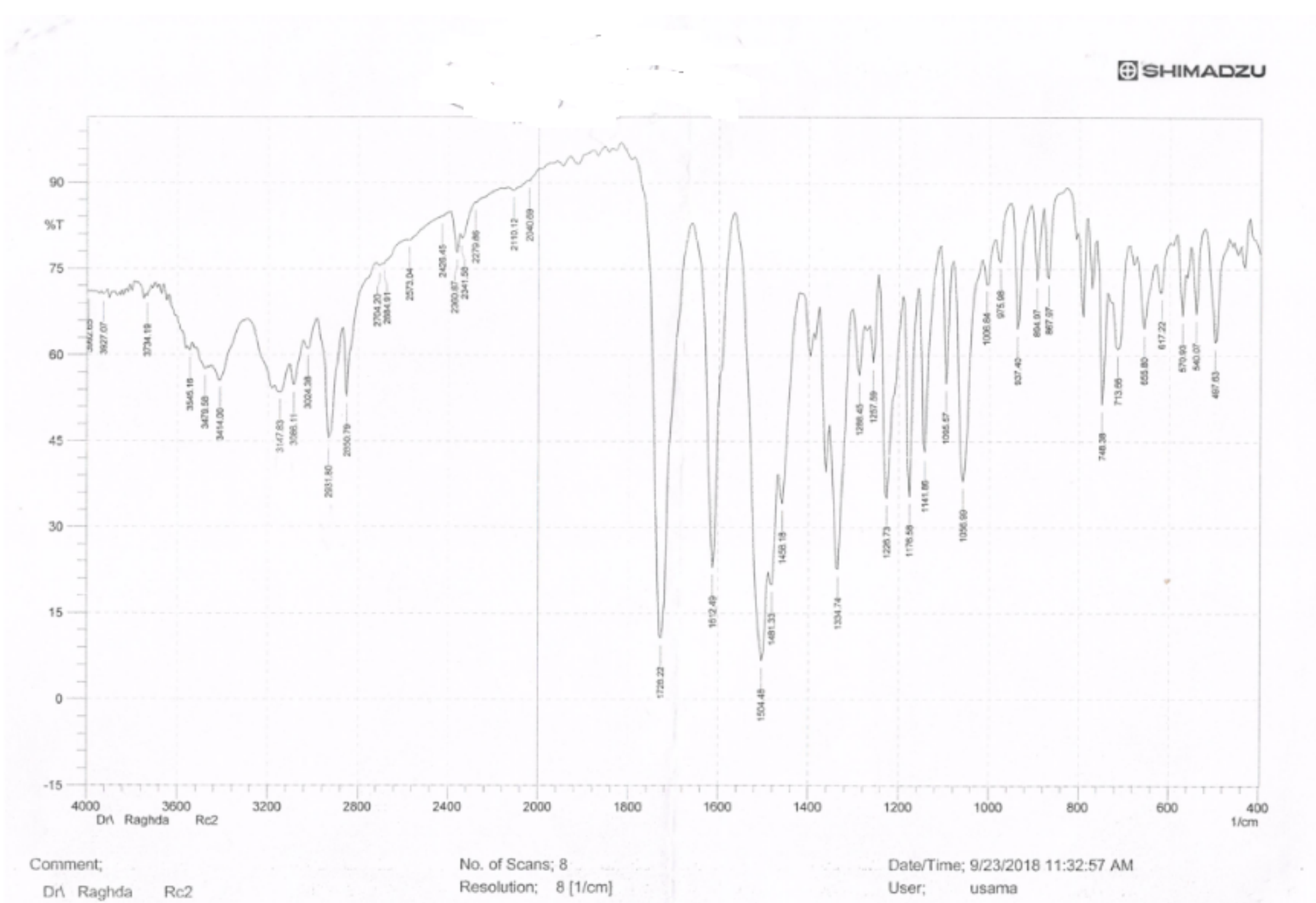

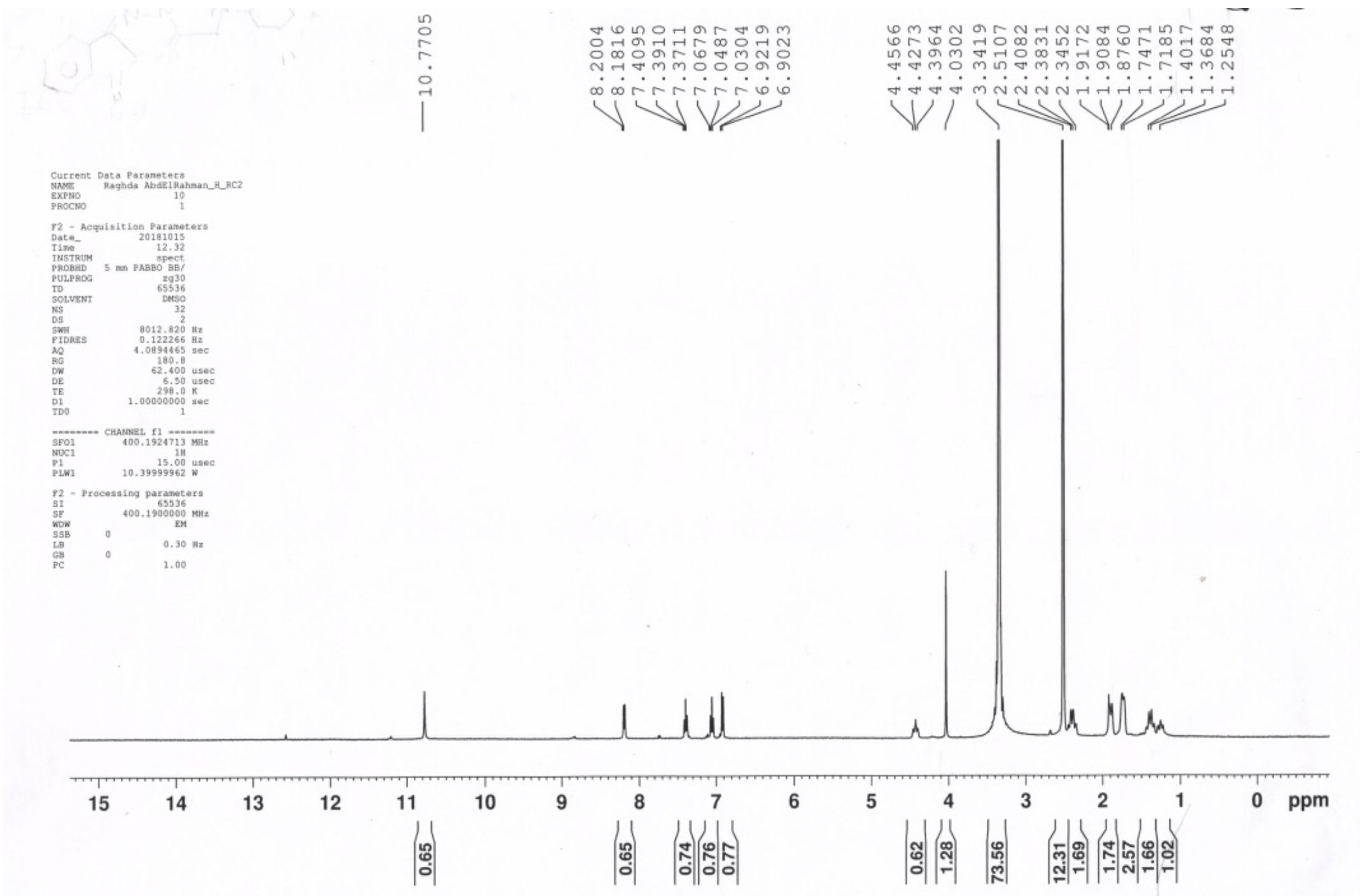
10.7705
8.2004
8.1816
7.4095
7.3910
7.3711
7.0679
7.0487
7.0304
6.9219
6.9023
4.4566
4.4273
4.3964
4.0302
3.3419
2.5107
2.4082
2.3831
2.3452
1.9172
1.9084
1.8760
1.7471
1.7185
1.4017
1.3684
1.2548
Current Data Parameters
NAME Raghda AbdElRahman_H_RC2
EXPNO 10
PROCNO 1
F2 - Acquisition Parameters
Date_ 20181015
Time 12.32
INSTRUM spect
PROBHD 5 mm PABBO BB/
PULPROG zg30
TD 65536
SOLVENT DMSO
NS 32
DS 2
SWH 8012.820 Hz
FIDRES 0.122266 Hz
AQ 4.0894465 sec
RG 180.8
DW 62.400 usec
DE 6.50 usec
TE 298.0 K
D1 1.00000000 sec
TD0 1
======== CHANNEL f1 ========
SFO1 400.1924713 MHz
NUC1 1H
P1 15.00 usec
PLW1 10.39999962 W
F2 - Processing parameters
SI 65536
SF 400.1900000 MHz
WDW EM
SSB 0
LB 0.30 Hz
GB 0
PC 1.00
15 14 13 12 11 10 9 8 7 6 5 4 3 2 1 0 ppm
0.65
0.65
0.74
0.76
0.77
0.62
1.28
73.56
12.31
1.69
1.74
2.57
1.66
1.02

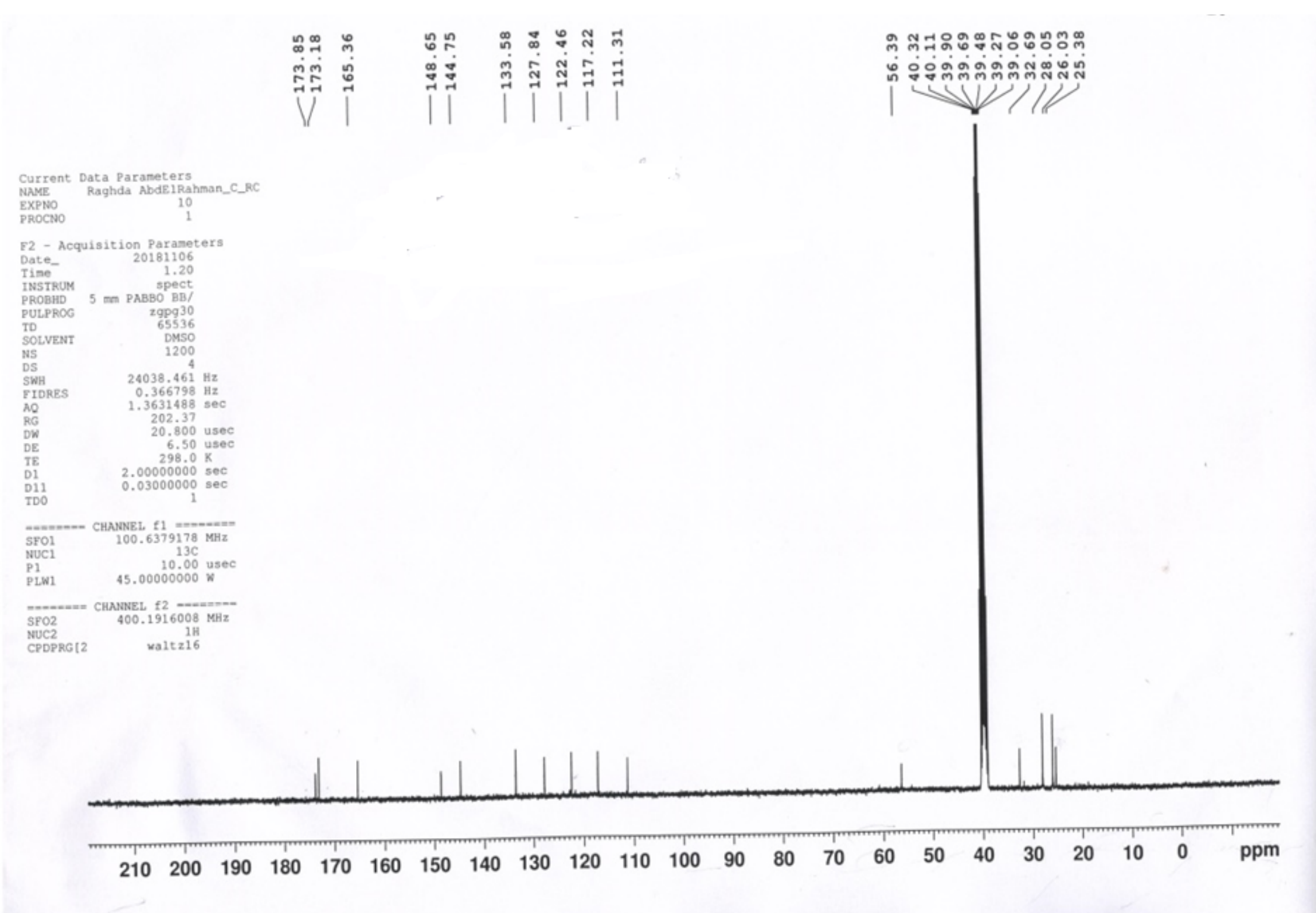
173.85
173.18
165.36
148.65
144.75
133.58
127.84
122.46
117.22
111.31
56.39
40.32
40.11
39.90
39.69
39.48
39.27
39.06
32.69
28.05
26.03
25.38
Current Data Parameters
NAME Raghda AbdElRahman_C_RC
EXPNO 10
PROCNO 1
F2 - Acquisition Parameters
Date_ 20181106
Time 1.20
INSTRUM spect
PROBHD 5 mm PABBO BB/
PULPROG zgpg30
TD 65536
SOLVENT DMSO
NS 1200
DS 4
SWH 24038.461 Hz
FIDRES 0.366798 Hz
AQ 1.3631488 sec
RG 202.37
DW 20.800 usec
DE 6.50 usec
TE 298.0 K
D1 2.00000000 sec
D11 0.03000000 sec
TD0 1
======== CHANNEL f1 ========
SFO1 100.6379178 MHz
NUC1 13C
P1 10.00 usec
PLW1 45.00000000 W
======== CHANNEL f2 ========
SFO2 400.1916008 MHz
NUC2 1H
CPDPRG[2 waltz16
210 200 190 180 170 160 150 140 130 120 110 100 90 80 70 60 50 40 30 20 10 0 ppm
